# Supplementary material for: Emergence of the primordial pre-60S from the 90S pre-ribosome
Source: Cell Rep. 2022 Apr 5;39(1):110640. doi: 10.1016/j.celrep.2022.110640 (PMC8994135; doi:10.1016/j.celrep.2022.110640)
Supplement: Document S1. Figures S1–S7 [file mmc1.pdf]

Cell Reports, Volume *39*

Supplemental information

Emergence of the primordial pre-60S from the 90S pre-ribosome

Sherif Ismail, Dirk Flemming, Matthias Thoms, José Vicente Gomes-Filho, Lennart Randau, Roland Beckmann, and Ed Hurt

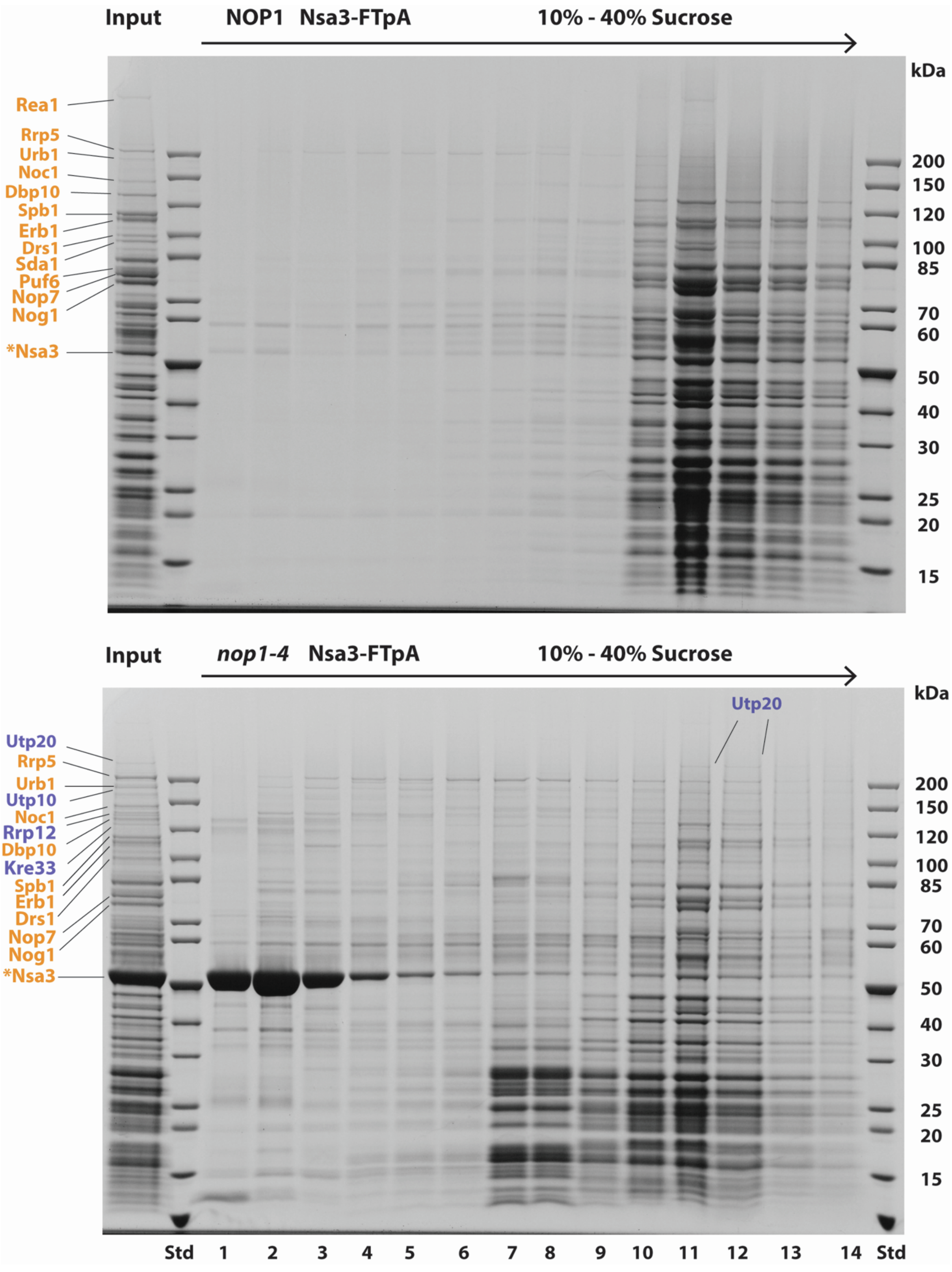

Figure S1. Sucrose Gradient Analysis of Nsa3 Pre-60S Particles from the *nop1-4* Mutant, Related to Figure 1

Affinity-purified Nsa3 particles from wild-type *NOP1* and *nop1-4* cells were fractionated by sucrose gradient centrifugation (10–40% sucrose) (w/v). 14 fractions were collected from each

gradient, which were TCA-precipitated and resuspended in SDS-sample buffer. The fractions were analyzed by 4–12% gradient SDS-PAGE and stained with Coomassie. Pre-60S assembly factors are colored in orange and 90S factors are colored in blue. The bait protein Nsa3 is indicated by an asterisk.

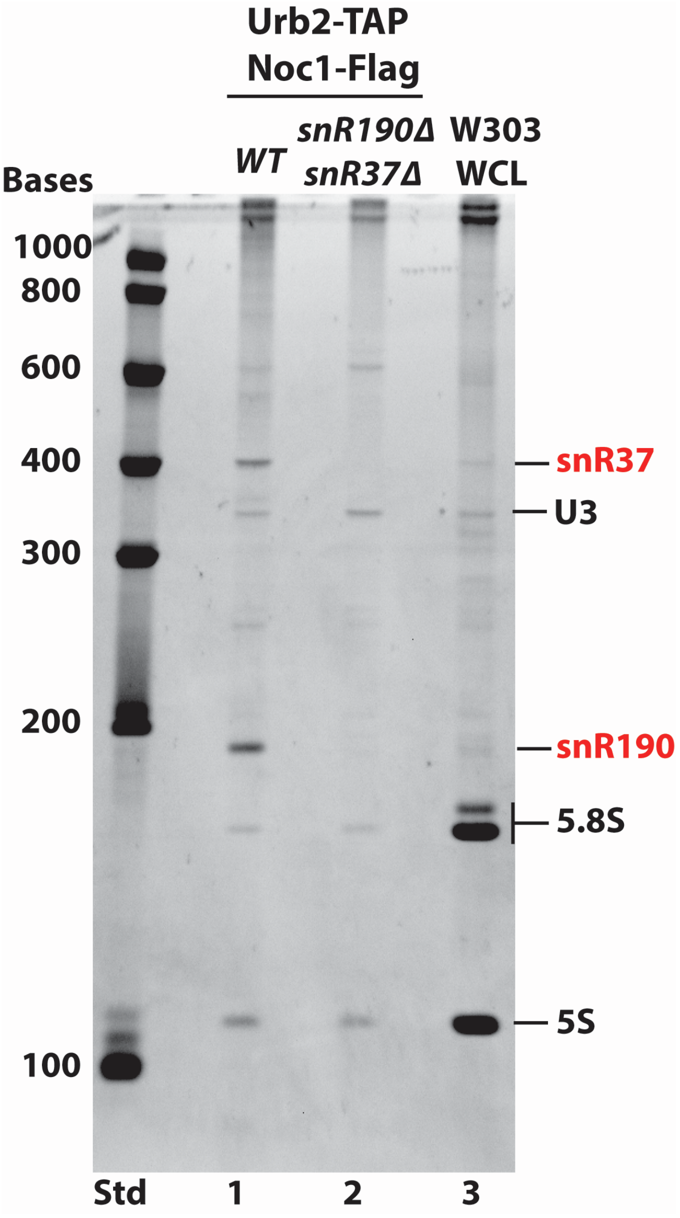

Figure S2. snR190 and snR37 are Absent from the Urb2–Noc1 Particle When Isolated from *snR190Δ snR37Δ* Double-Deletion Strain, Related to Figure 3 and 4

Polyacrylamide/urea gel electrophoresis and SYBR Green staining of RNA extracted from the final eluates of affinity-purified Urb2-TAP–Noc1-Flag, derived from the wild-type or *snR190Δ snR37Δ* double-deletion strains. RNA of a whole cell lysate (WCL) from strain W303 was also loaded on the 8% polyacrylamide/urea gel, which was stained with SYBR Green to detect the snR37 and snR190 bands, which are indicated in red on the right.

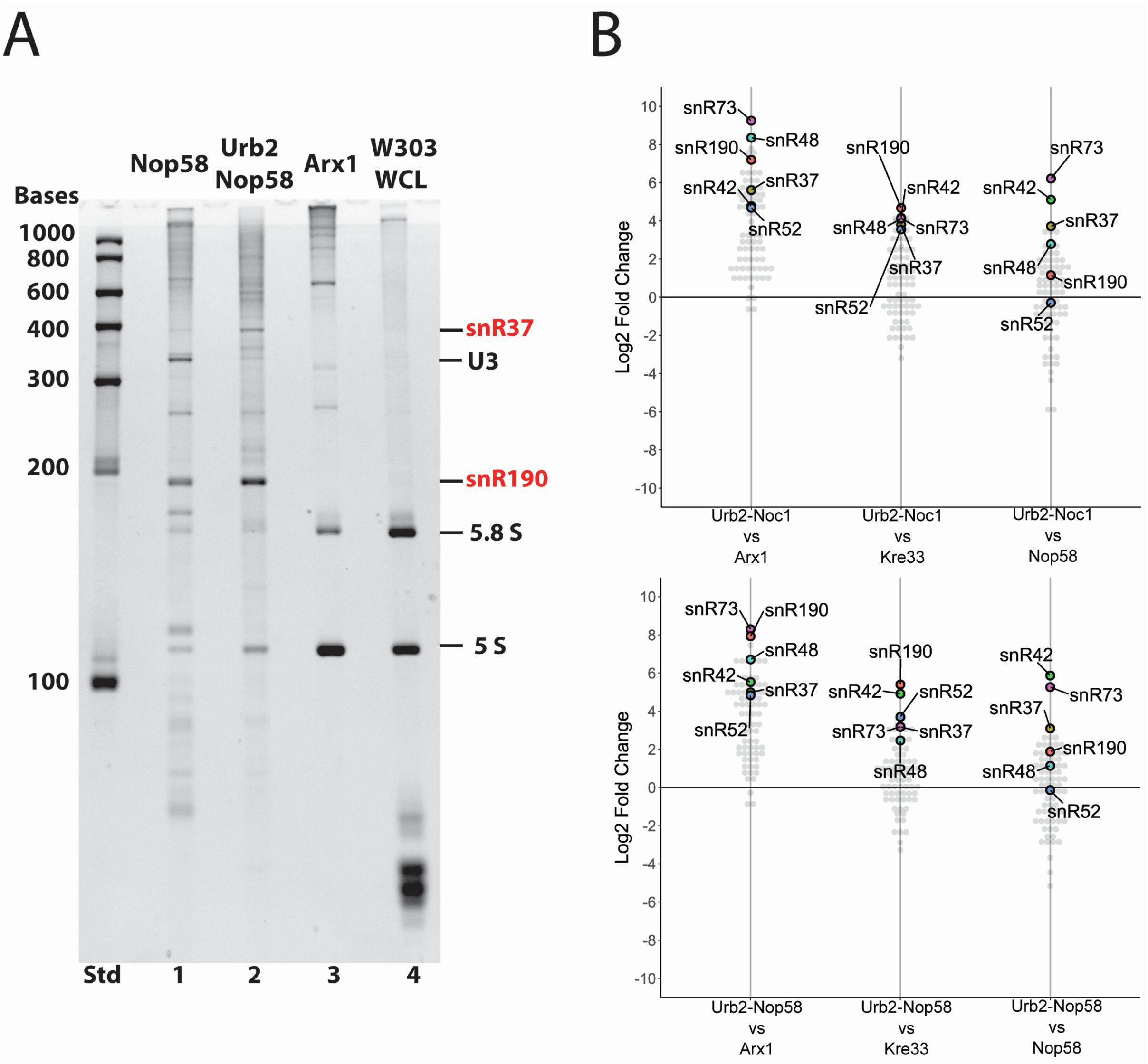

Figure S3. Identification of the snoRNAs Co-enriched in the Affinity-Purified Primordial Pre-60S Particles, Related to Figure 3

(A) SYBR Green staining of the 8% polyacrylamide/urea gel, on which was separated RNA extracted from Nop58-FTpA, Urb2-TAP–Nop58-Flag, and Arx1-FTpA affinity-purified particles, in comparison to total RNA from whole-cell lysate (WCL) of the wild-type W303 strain. Nop58 was used to reveal the pool of C/D box snoRNAs, whereas Urb2–Nop58 was used to show the snoRNA pool enriched in the primordial pre-60S.

(B) Illumina RNA sequencing of RNA extracted from Urb2-TAP Noc1-Flag, Urb2-TAP Nop58-Flag, Nop58-FTpA, Kre33-FTpA, and Arx1-FTpA final eluates. The Log2 Fold change of Illumina read counts was calculated for Urb2-Noc1 and Urb2-Nop58 preparations in comparison to Arx1, Kre33 and Nop58 samples (see Table S3 for further details).

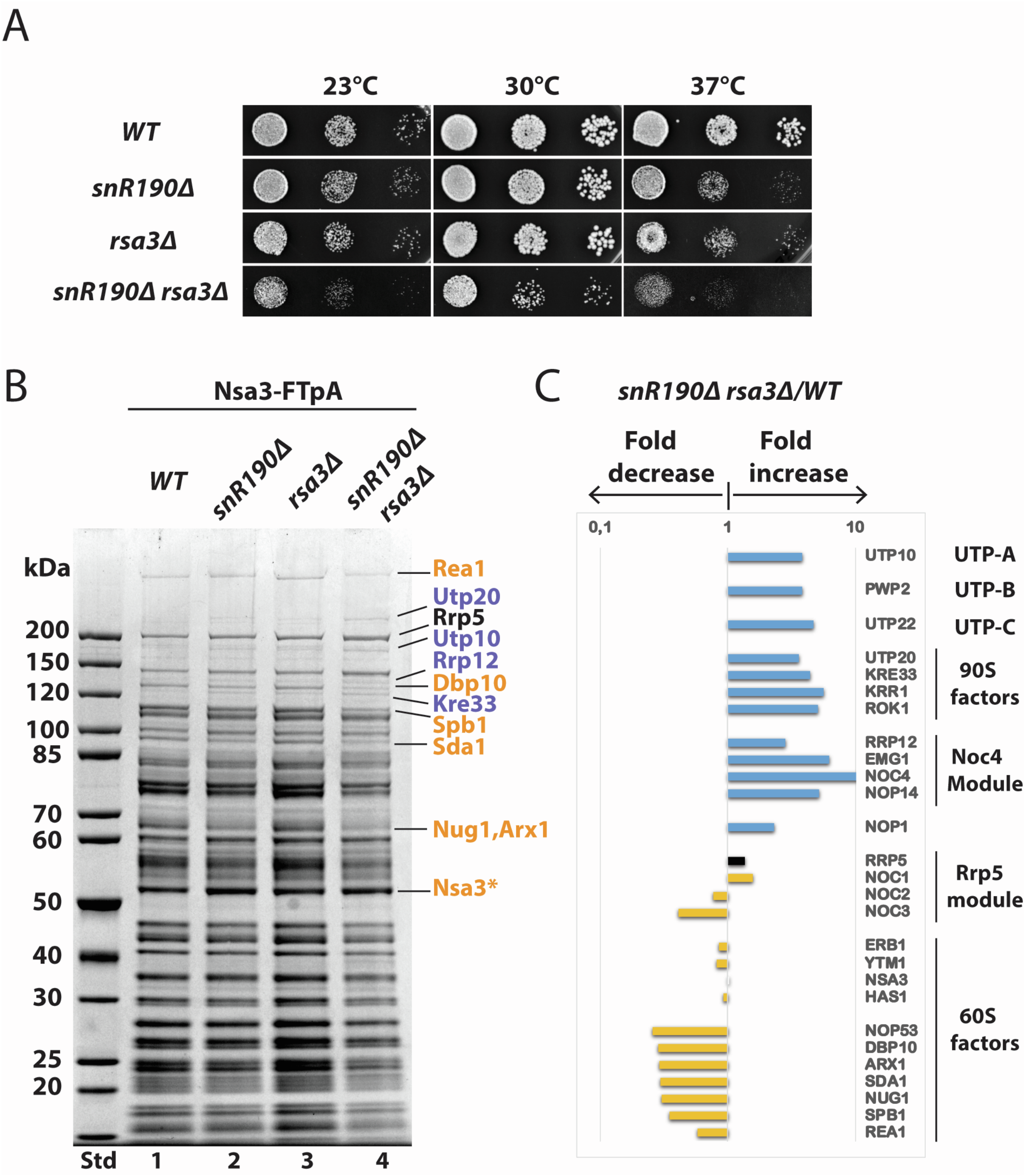

Figure S4. Synthetically Enhanced Growth Defect between snR190 and the Urb1 Module Factor Rsa3, Related to Figure 4

(A) Dot-spot growth analysis of wild-type W303 (WT), *snR190Δ*, *rsa3Δ*, and *snR190Δ rsa3Δ* strains. Tenfold serial dilutions were spotted onto YPD plates. The plates were incubated at 23, 30, or 37°C for 2 days.

(B) Affinity purification of Nsa3-FTpA from wild-type W303, *snR190Δ*, *rsa3Δ*, and *snR190 Δ rsa3Δ* strains. Flag eluates were analyzed on a 4–12% gradient SDS-PAGE gel and stained with Coomassie. The major bands labeled on the right were identified by mass spectrometry.

The 90S factors are colored in blue and pre-60S factors are colored in orange. The bridging factor Rrp5 is colored in black. The Nsa3 bait is indicated by an asterisk.

(C) Semiquantitative mass spectrometry analysis of the Nsa3-FTpA eluates in panel B. The label-free quantification values (normalized to Nsa3) derived from the *snR190Δ rsa3Δ* strain were divided by those obtained for the WT particles, which represents fold change in 90S (blue bars) and pre-60S (orange bars) assembly factors. For the whole data set of the mass spectrometry analysis, see Table S5.

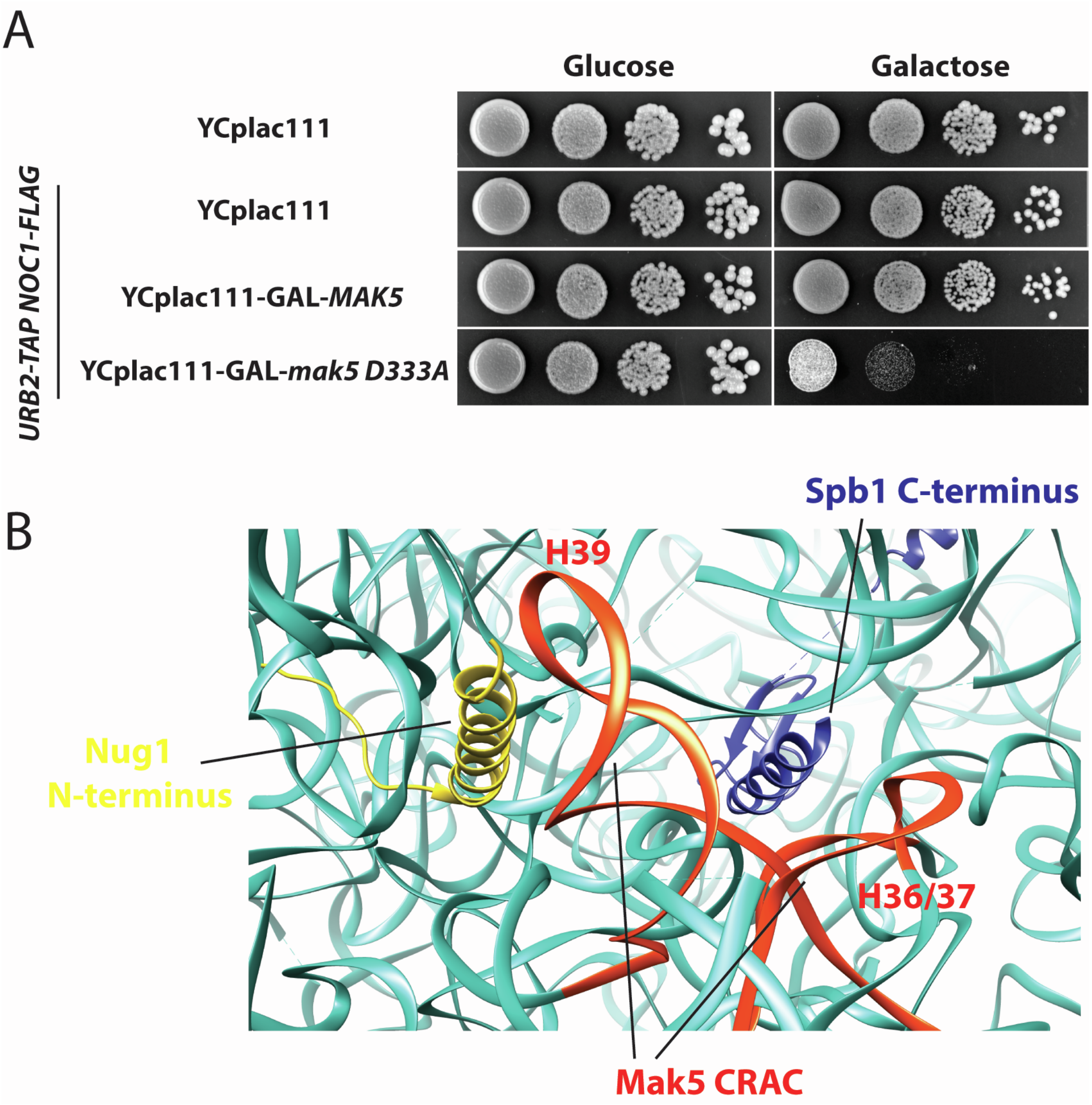

Figure S5. Dominant-Negative Mak5 D333A Walker B Mutant and Mak5-Binding Site in the Pre-60S Particles, Close to the N Terminus of Nug1 and the C Terminus of Spb1, Related to Figure 6

(A) Dot-spot growth analysis of the dominant-negative phenotype of *mak5 D333A* overexpression in the Urb2-TAP Noc1-Flag background. Tenfold serial dilutions of the indicated cells were spotted onto SDC−Leu (glucose) and SGC−Leu (galactose) plates. The plates were incubated at 30°C for 3 days.
(B) Nsa1–Ytm1 cryo-EM structure state D (PDB ID: 6EM5) with indicated CRAC crosslinking sites of Mak5 (Brüning et al., 2018) at helices H36/37 and H39 (Red). In addition, the nearby Nug1 N terminus (yellow) and Spb1 C terminus (blue) are highlighted.

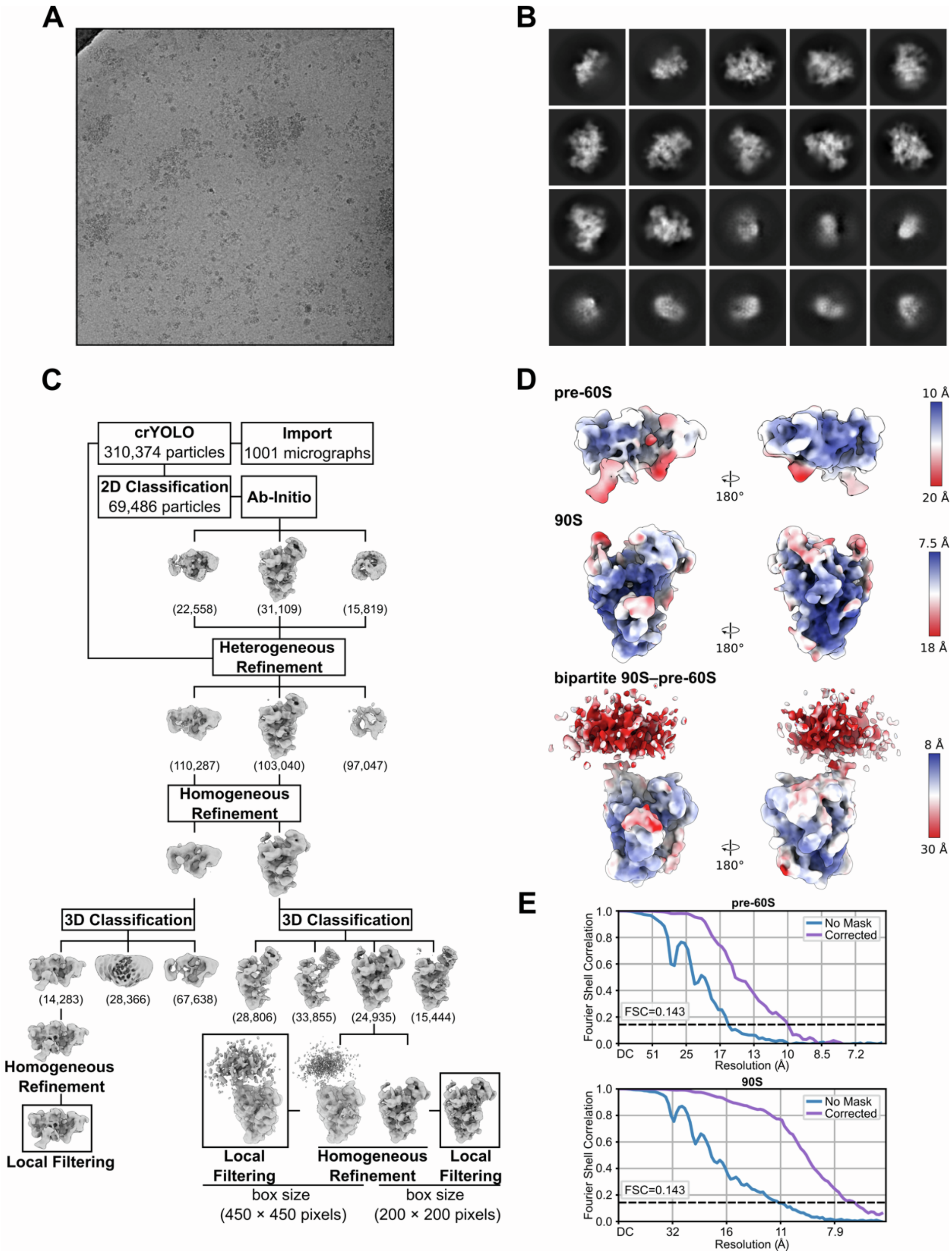

Figure S6. Sorting Scheme for the Urb2–Noc1 *mak5* D333A Particles, Related to Figure 7

(A, B) Representative electron micrograph (A) and selected 2D classification averages of the Urb2–Noc1 preparation isolated from the dominant-negative *mak5* D333A mutant (B).
(C) Cryo-EM processing scheme. Particle numbers are indicated in parentheses.
(D) The final 3D reconstructions after local filtering and colored according to their local resolution.
(E) Fourier shell correlation curves of the final pre-60S and 90S reconstructions. The FSC thresholds at 0.143 are indicated as dashed lines.

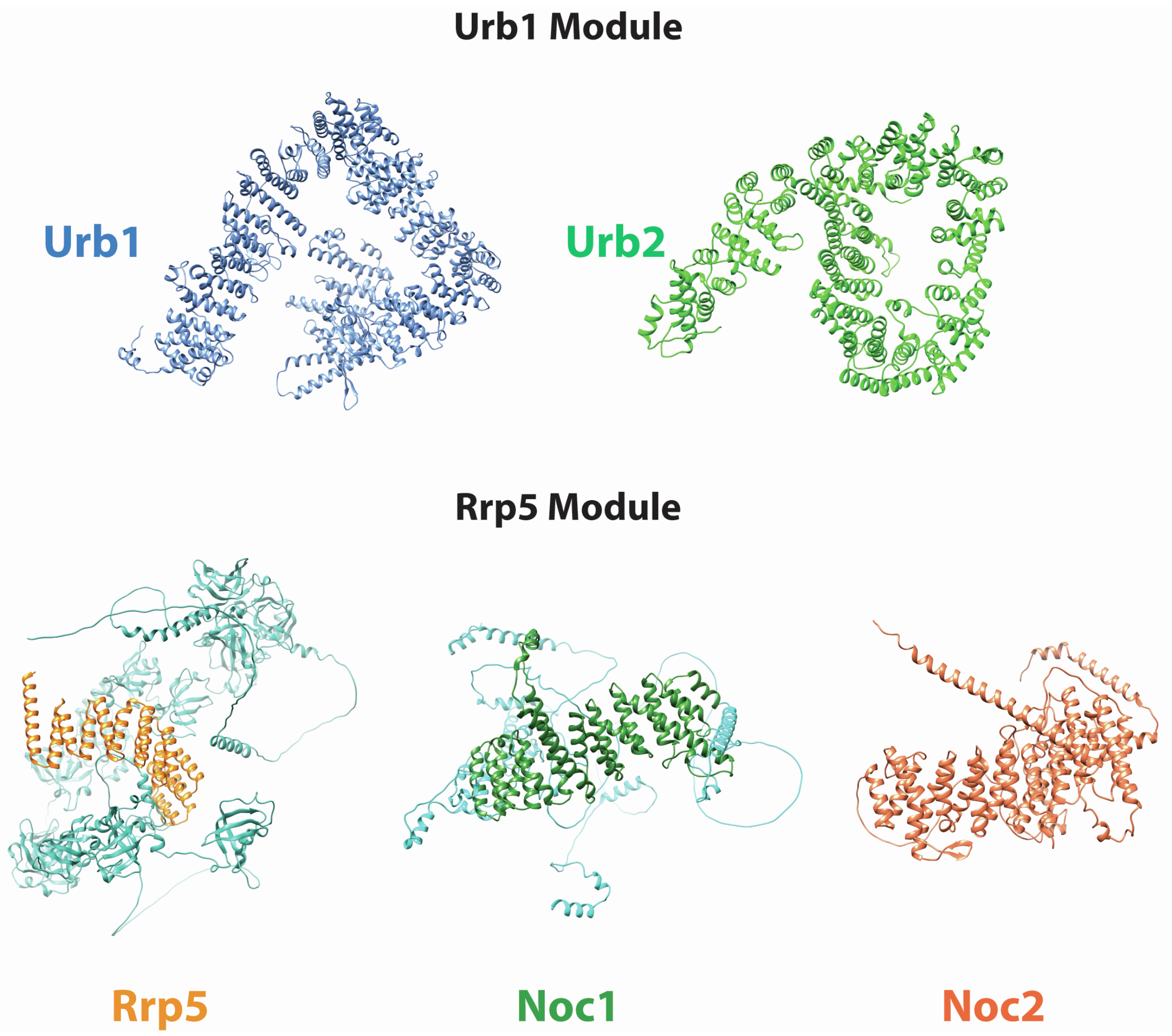

Figure S7. Structure prediction of Urb1 and Rrp5 module members, Related to Figure 7

Structure prediction of ribosome assembly factors of the Urb1 and Rrp5 modules using AlphaFold (Jumper et al., 2021). The α-helical repeats of Urb1 (light blue; code number: P34241), Urb2 (light green, code number: P47108), Rrp5 (orange, code number: Q05022), Noc1 (dark green, code number: Q12176) and Noc2 (coral, code number: P39744) forming α-solenoid folds are depicted. Beside the α-helical repeats in the C-terminal half (orange), Rrp5 contains 12 S1-like domains in row in its N-terminal part (greenish blue).
