# Supplementary material for: Emergence of the primordial pre-60S from the 90S pre-ribosome
Source: Cell Rep. 2022 Apr 5;39(1):110640. doi: 10.1016/j.celrep.2022.110640 (PMC8994135; doi:10.1016/j.celrep.2022.110640)
Supplement: Document S2. Article plus supplemental information [file mmc8.pdf]

Cell Reports

Resource

Emergence of the primordial pre-60S from the 90S pre-ribosome

Graphical abstract

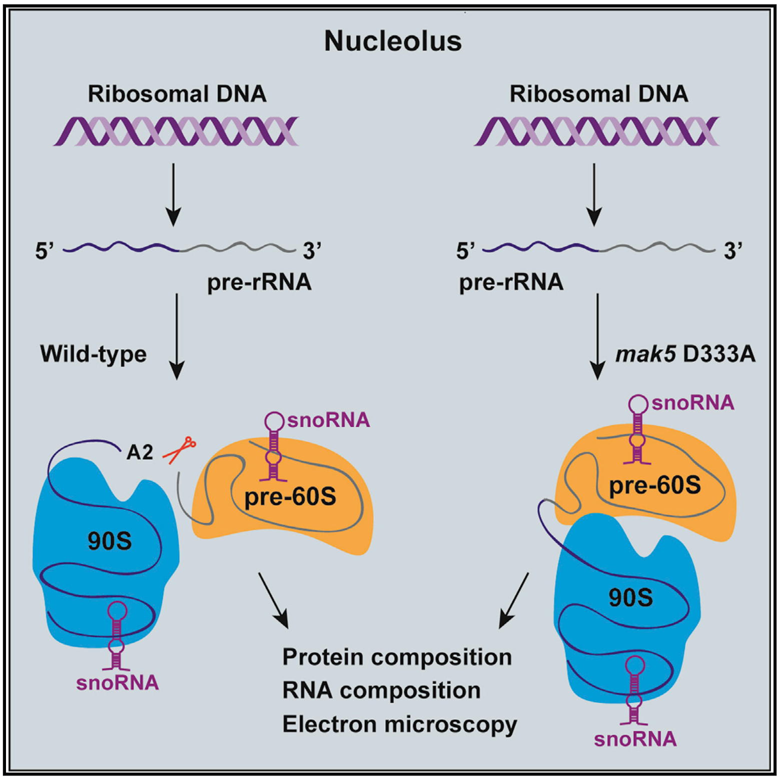

Highlights

- Affinity purification of primordial pre-60S particle via split-tag method
- Primordial pre-60S contains specific snoRNAs and assembly factors
- Arrest of 90S-pre-60S pre-ribosome in *mak5* helicase mutant
- 90S-pre-60S pre-ribosome exhibits a bipartite structure

Authors

Sherif Ismail, Dirk Flemming, Matthias Thoms, José Vicente Gomes-Filho, Lennart Randau, Roland Beckmann, Ed Hurt

Correspondence

beckmann@genzentrum.lmu.de (R.B.),
ed.hurt@bzh.uni-heidelberg.de (E.H.)

In brief

Ismail et al. report the isolation of a primordial pre-60S particle, composed of a specific set of assembly factors and snoRNAs. This assembly intermediate is visualized by cryo-EM while emerging from the 90S pre-ribosome, revealing a 90S-pre-60S bipartite structure.

Ismail et al., 2022, Cell Reports *39*, 110640
April 5, 2022 © 2022 The Author(s).
https://doi.org/10.1016/j.celrep.2022.110640

CellPress

Cell Reports

CellPress
OPEN ACCESS

Resource

Emergence of the primordial pre-60S from the 90S pre-ribosome

Sherif Ismail,[1] Dirk Flemming,[1] Matthias Thoms,[2] José Vicente Gomes-Filho,[3] Lennart Randau,[3] Roland Beckmann,[2,*] and Ed Hurt[1,4,*]
[1]Heidelberg University Biochemistry Center (BZH), Im Neuenheimer Feld 328, 69120 Heidelberg, Germany
[2]Gene Center, Ludwig-Maximilians-Universität München, Feodor-Lynen-Straße 25, 81377 Munich, Germany
[3]Philipps-Universität Marburg, Karl-von-Frisch-Str. 8, 35043 Marburg, Germany
[4]Lead contact
*Correspondence: beckmann@genzentrum.lmu.de (R.B.), ed.hurt@bzh.uni-heidelberg.de (E.H.)
https://doi.org/10.1016/j.celrep.2022.110640

SUMMARY

Synthesis of ribosomes begins in the nucleolus with formation of the 90S pre-ribosome, during which the pre-40S and pre-60S pathways diverge by pre-rRNA cleavage. However, it remains unclear how, after this uncoupling, the earliest pre-60S subunit continues to develop. Here, we reveal a large-subunit intermediate at the beginning of its construction when still linked to the 90S, the precursor to the 40S subunit. This primordial pre-60S is characterized by the SPOUT domain methyltransferase Upa1-Upa2, large α-solenoid scaffolds, Mak5, one of several RNA helicases, and two small nucleolar RNA (snoRNAs), C/D box snR190 and H/ACA box snR37. The emerging pre-60S does not efficiently disconnect from the 90S pre-ribosome in a dominant *mak5* helicase mutant, allowing a 70-nm 90S-pre-60S bipartite particle to be visualized by electron microscopy. Our study provides insight into the assembly pathway when the still-connected nascent 40S and 60S subunits are beginning to separate.

INTRODUCTION

Protein synthesis in living cells requires functional ribosomes, which are conserved in all domains of life. In eukaryotic cells, the formation of the 60S and 40S ribosomal subunits occurs through an extremely energy-consuming and highly conserved cascade of synthesis, assembly, and maturation steps. This has been well studied in the yeast *Saccharomyces cerevisiae*, in which ribosomal RNA (rRNA) is first transcribed in the nucleolus from ribosomal DNA repeats by RNA polymerase I to produce a polycistronic transcript. This precursor, called the 35S pre-rRNA (47S pre-rRNA in human), contains both external (5′ ETS and 3′ ETS) and internal (ITS1 and ITS2) transcribed spacer sequences, which flank the mature 18S rRNA (part of the 40S), and the 5.8S and 25S rRNAs (parts of the 60S subunit). 5S rRNA is transcribed separately by RNA polymerase III and, together with Rpl5 (uL18) and Rpl11 (uL5), this nascent 5S ribonucleoprotein (RNP) is incorporated into the early pre-60S subunit in a subsequent step, the details of which remain unclear. Initially, ribosome assembly proceeds through a number of endonucleolytic RNA cleavages, followed by RNA modification and folding reactions of the nascent pre-rRNA and recruitment of ribosomal proteins. All these steps are facilitated by a myriad of ribosome assembly factors, which sequentially associate with the evolving pre-ribosomal particles and drive the whole process from the nucleolus into the cytoplasm (reviewed in Baßler and Hurt, 2019; Woolford and Baserga, 2013).

Already during transcription from the ribosomal DNA (rDNA), a first set of early ribosome assembly factors associate with the emerging nascent pre-rRNA to gradually form the 90S pre-ribosome (SSU processome), which is the first stable pre-ribosomal assembly intermediate that can be biochemically isolated (Dragon et al., 2002; Grandi et al., 2002) and structurally analyzed (Chaker-Margot et al., 2017; Cheng et al., 2020; Kornprobst et al., 2016; Singh et al., 2021; Sun et al., 2017). Several of the 90S assembly factors form modules, which are incorporated into the evolving 90S particle in a hierarchical order (Baßler et al., 2017; Hunziker et al., 2016; Krogan et al., 2004; Pérez-Fernández et al., 2007). In particular, the emerging 5′ ETS forms a seed onto which UTP-A, UTP-B, and U3 small nucleolar RNP (snoRNP) modules are recruited, whereas emergence of the nascent 18S rRNA subdomains facilitates the incorporation of further modules (e.g., Mpp10-Imp3-Imp4-Sas10, UTP-C, Bms1-Rcl1, Kre33-Bfr2-Enp2-Lcp5, Noc4-Nop14-Emg1-Rrp12-Enp1) and assembly factors (e.g., Utp20, Dhr1, Rrp5), which all together eventually form the 90S pre-ribosome (Chaker-Margot et al., 2015; Cheng et al., 2019; Hunziker et al., 2019; Sá-Moura et al., 2017; Zhang et al., 2016). After dismantling of the 5′ ETS together with most of the 90S factors, which depends on a separating endo-nucleolytic cleavage within ITS1 at site A_2, the primordial pre-40S emerges (Cheng et al., 2020), which continues to develop until export into the cytoplasm, where final maturation occurs. Here, a specific quality control step involving the interaction of the pre-40S particle with the 60S subunit tests the still nascent small subunit for its

© 2022 The Author(s).
This is an open access article under the CC BY license (http://creativecommons.org/licenses/by/4.0/).

functionality in a translation-like cycle, accompanied by final Nob1 endonuclease-mediated 20S>18S rRNA processing and residual 40S assembly factor removal (Strunk et al., 2012; Lamanna and Karbstein, 2009; Pertschy et al., 2009; Strunk et al., 2012).

During the earliest steps of ribosome assembly when the nascent pre-rRNA is cleaved within ITS1, the small- and large-subunit assembly pathways already begin to separate (Udem and Warner, 1972). However, the co-transcriptional cleavage within ITS1 does not occur directly after the emergence of ITS1, but is delayed until 5.8S rRNA, ITS2, and the 5′ part of the 25S rRNA have been transcribed (Axt et al., 2014; Koš and Tollervey, 2010; Osheim et al., 2004). There is evidence that Rrp5, a common pre-ribosomal factor linking the nascent pre-40S (i.e., the 90S pre-ribosome) with the emerging pre-60S, binds at the ITS1 region, thereby coordinating these processes (Khoshnevis et al., 2019; Lebaron et al., 2013; Venema and Tollervey, 1996). Following pre-rRNA cleavage at site A_2, Rrp5 is thought to detach from the 90S pre-ribosome but remain bound to the nascent pre-60S along with Noc1 and Noc2, which together form the Rrp5-Noc1-Noc2 module (Hierlmeier et al., 2013). However, there is still a gap of knowledge regarding the pre-60S particles at this very early stage. These particles are only ill-defined but contain another chaperone module of unknown function, composed of Urb1/Npa1, Urb2/Npa2, Dbp6, Nop8, and Rsa3 (Dez et al., 2004; Joret et al., 2018; Mnaimneh et al., 2004; Rosado and De La Cruz, 2004; Rosado et al., 2007). The $27SA_2$ pre-rRNA, which is the predominant form in these early particles, is then trimmed to generate the 27SB pre-rRNA and the derived nucleolar pre-60S particles could be elucidated by cryoelectron microscopy (cryo-EM), which revealed the initial folding of rRNA domains and the associated assembly factors together with the ribosomal proteins (Kater et al., 2017; Sanghai et al., 2018; Zhou et al., 2018). These particles showed that the solvent-exposed side of the large subunit is formed by compaction of the 5.8S rRNA, ITS2, and the 25S rRNA domains I and II in order to form a stable core for further assembly, where the 25S rRNA domain VI is next assembled forming a tight ring-like structure (Kater et al., 2017; Sanghai et al., 2018; Zhou et al., 2018).

With the departure of the pre-60S subunit from the nucleolus into the nucleoplasm, several remodeling events occur, which involve the AAA-type Rea1 ATPase, which in a first round removes the Ytm1-Erb1 complex from the pre-60S (Baßler et al., 2010; Kater et al., 2017). In a second Rea1 step, which requires the Rix1-Ipi1-Ipi3 complex, Rsa4 is removed from the pre-60S particle in the nucleoplasm (Ulbrich et al., 2009), during which also Rpf2-Rrs1 dissociate, allowing rotation of the 5S RNP to adopt its mature position (Barrio-Garcia et al., 2016). Another major maturation event on the nucleoplasmic pre-60S particles is the removal of the prominent foot structure, which requires ITS2 processing and dismantling of the foot-associated assembly factors such as Nsa3/Cic1, Nop7, Nop15, and Rlp7 (Allmang et al., 1999; Gasse et al., 2015; Mitchell et al., 1997; Thoms et al., 2015; Thomson and Tollervey, 2010). After these massive remodeling events, the nuclear export factor Nmd3 is recruited to the pre-60S particles, which allows transport of the nascent large subunit into the cytoplasm (Ma et al., 2017; Matsuo et al., 2014). In this subcellular compartment, the final maturation of the pre-60S takes place, which includes release of the remaining assembly factors and proofreading of the PTC.

Another key process during the assembly of pre-ribosomal particles is rRNA base and ribose modification. Both covalent modifications occur co-transcriptionally as well as post-transcriptionally (Koš and Tollervey, 2010), but it is thought that the rRNA must be *in status nascendi*, that is, not yet compactly folded, so that the modification machineries can reach nucleotide sites that are later buried within the compactly folded mature rRNA. The vast majority of known rRNA modifications are catalyzed by small nucleolar RNA (snoRNA)-guided RNP complexes, of which there are two classes, the C/D box and H/ACA box snoRNAs, which form specific snoRNPs characterized by their core factors (Balakin et al., 1996; Ganot et al., 1997; Kiss-László et al., 1996; Reichow et al., 2007). C/D box snoRNPs are responsible for 2′-*O*-ribose methylation and contain the methyltransferase Nop1 (fibrillarin in human) and the core factors Nop58, Nop56, and Snu13. H/ACA snoRNPs are responsible for pseudouridylation, for which Cbf5 (dyskerin in human) acts as a pseudouridine synthase along with the core factors Nhp2, Gar1, and Nop10 (Reichow et al., 2007; Watkins and Bohnsack, 2012). Interestingly, some snoRNAs play an essential structural role during ribosome synthesis. This role is biochemically and structurally well understood in the case of the C/D box U3 snoRNP, which is an intrinsic module of the 90S pre-ribosome, essential for 90S pre-rRNA processing and maturation (Chaker-Margot et al., 2017; Cheng et al., 2020; Kornprobst et al., 2016). At present, however, it is unclear whether pre-60S ribosomes require specific snoRNAs for their assembly.

To shine further light on the very early 60S assembly pathway and find out whether specific snoRNPs play a role in this process, we performed biochemical and genetic analyses, with a first focus on a specific mutant allele of *NOP1* (*nop1-4*) that encodes a core factor of the C/D box snoRNAs (Tollervey et al., 1993). Based on findings obtained with *nop1-4*, we could perform specific split-tag affinity-purifications that led to the identification of a primordial pre-60S particle containing the C/D box snR190 and the H/ACA box snR37. Prompted by these findings, we exploited a dominant *mak5* helicase mutant, which hindered detachment of the 90S small-subunit pre-ribosome from the primordial pre-60S during ribosome biogenesis. This situation enabled us to use electron microscopy (EM) to visualize the long-sought particle formed between the nascent small (90S) and large subunit (pre-60S), giving insight into how the pre-40S and pre-60S assembly pathways begin to diverge.

RESULTS

Pre-60S particles from the *nop1-4* mutant entrap 90S factors

It is unclear whether the earliest pre-60S particles co-assemble with snoRNPs in a manner similar to that observed for the 90S small-subunit pre-ribosome, which carries the U3 snoRNP as an integrated structural module. Despite this uncertainty, we recalled an old observation that a specific mutant allele of *NOP1* (*nop1-4*; Ala245>Val), which encodes an essential subunit of the U3 snoRNP that is also a core factor of the many other C/D box

CellPress
OPEN ACCESS

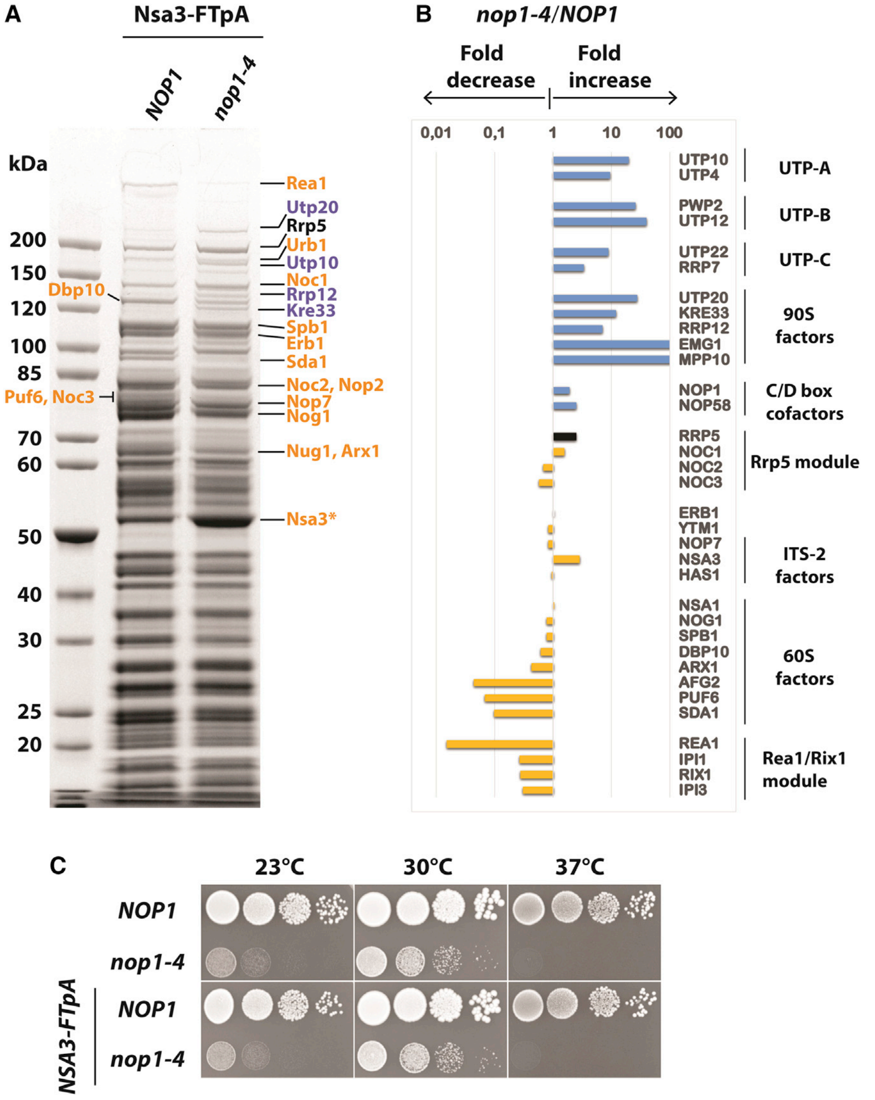

Figure 1. Comparison of Nsa3 particles isolated from wild-type *NOP1* and *nop1-4* mutant cells

(A) Nsa3-FTpA (FLAG-TEV-ProtA) was affinity purified from wild-type and *nop1-4* cells. Final eluates were analyzed on a 4%–12% gradient SDS-polyacrylamide gel stained with Coomassie blue. The major bands labeled on the side were identified by mass spectrometry. The 90S assembly factors are colored in blue and pre-60S factors are colored in orange. The Nsa3 (Cic1) bait is indicated by an asterisk.

(B) Semi-quantitative mass spectrometry analysis of the Nsa3-FTpA eluates in (A). The label-free quantification values (normalized to Erb1) derived from the *nop1-4* mutant particles were divided by those obtained for the wild-type particles (*NOP1*), which represents fold change in 90S (blue bars) and pre-60S (orange bars) assembly factors. The bar of the bridging factor Rrp5 is shown in black. For the whole dataset of the mass spectrometry analysis, see Table S1.

(C) Dot-spot growth analysis of *nop1-4* and isogenic *NOP1* cells either not containing (top) or carrying (bottom) chromosomally integrated *Nsa3*-FTpA. The 10-fold serial dilutions of the cells were spotted on YPD (yeast extract peptone dextrose) plates and incubated at 23°C, 30°C, or 37°C for 3 days.

snoRNAs (Balakin et al., 1996; Schimmang et al., 1989), induced the formation of abnormal pre-60S particles, which exhibited a disturbed sedimentation of rRNA and ribosomal large-subunit proteins on sucrose gradients (Tollervey et al., 1993). To explain this 60S defect on a molecular basis, we affinity purified Nsa3 (also known as Cic1), a pre-60S factor associated with a broad range of nascent 60S subunits from early nucleolar to intermediate nuclear stages, from both wild-type and *nop1-4* mutant cells (Figure 1). Unexpectedly, among the typical pre-60S factors, we detected many 90S factors co-enriched in the *nop1-4* mutant but not wild-type pre-60S particles (Figures 1A and 1B; Table S1). By contrast, assembly factors part of later large-subunit intermediates such as the Rea1-Rix1 complex and Sda1 were decreased in the Nsa3 eluate of the *nop1-4* mutant (Figures 1A and 1B; Table S1). Since *nop1-4* cells exhibit a slow growth phenotype at 30°C, the permissive temperature applied to isolate the pre-60S particles, we confirmed that the FLAG-TEV-protA (FTpA) tag on the Nsa3 did not exaggerate the growth defect (Figure 1C). Sucrose gradient centrifugation of Nsa3-associated particles from both *NOP1* and *nop1-4* cells did not show a significant change in sedimentation of the particles, although a free pool of Nsa3 no longer associating with pre-60S became evident (Figure S1). We do not know the reason for this free Nsa3, but the correct recycling of Nsa3 during the disturbed pre-60S biogenesis could have caused this abnormality. Collectively, these findings suggest that the specific *nop1-4* mutation shifts the Nsa3-derived pre-60S particles to an earlier stage, explaining in retrospect our initial findings of impaired 60S biogenesis in the *nop1-4* strain (Tollervey et al., 1993).

Pre-60S particles affinity purified via Urb2-Noc1 carry specific C/D box and H/ACA snoRNPs

Although the *nop1-4* mutation might have caused a 90S biogenesis defect before a pleiotropic feedback on pre-60S formation developed, we also considered another scenario: that *nop1-4* directly inhibited pre-60S assembly at an early stage, possibly due to a 60S-specific C/D box snoRNP (or snoRNPs) carrying the mutated Nop1 Ala245>Val. Hence, we searched for snoRNP-containing pre-60S particles that precede the classical and structurally well-characterized nucleolar pre-60S particles, which are all devoid of snoRNPs (Kater et al., 2017; Sanghai et al., 2018; Zhou et al., 2018). For this purpose, we used a split-tag affinity purification method using Urb2 as first bait and Noc1 as second. Noc1 was chosen for its

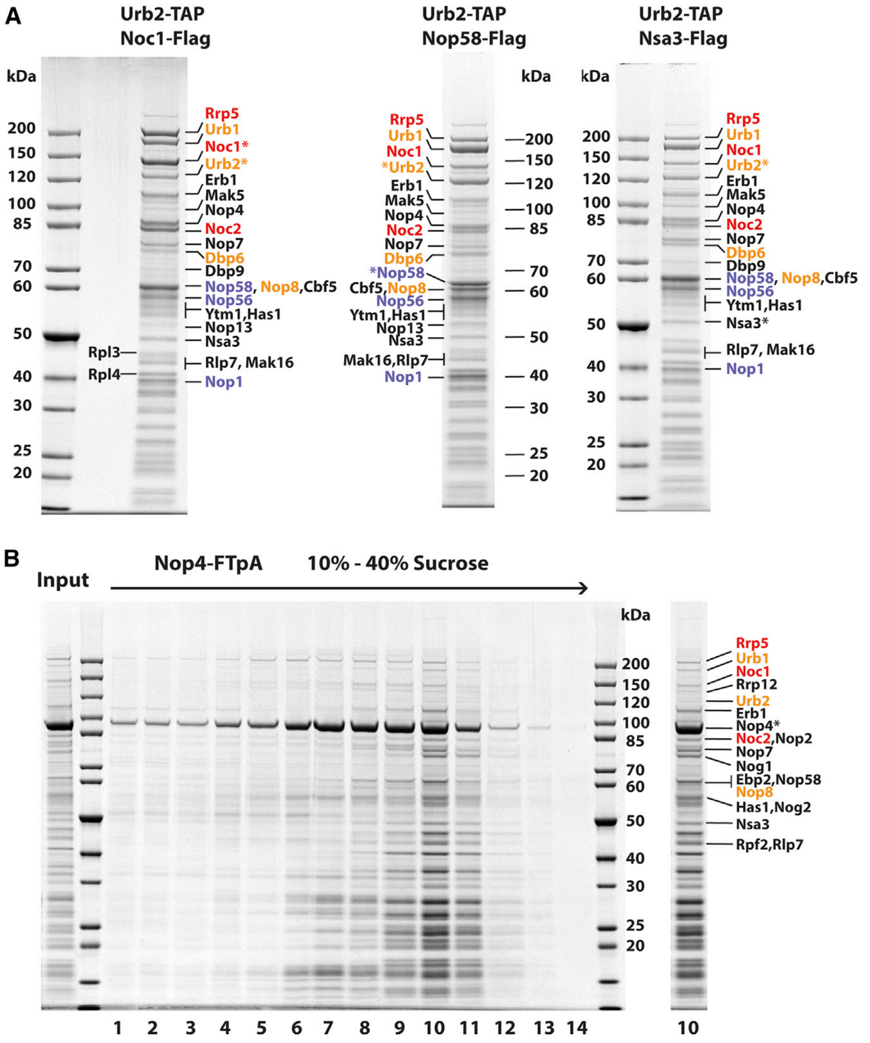

Figure 2. Split-tag affinity purification of primordial pre-60S particles via different baits exhibiting a unique pattern of assembly factors and snoRNPs

(A) Split-tag affinity purification of Urb2-TAP-Noc1-FLAG, Urb2-TAP-Nop58-FLAG, and Urb2-TAP-Nsa3-FLAG pre-ribosomal particles. The final FLAG eluates were analyzed on a 4%–12% gradient SDS-PAGE gel stained with Coomassie blue. The major bands labeled on the side were identified by mass spectrometry. Rrp5 module factors are colored in red, Urb1 module factors in orange, and C/D box snoRNA cofactors in blue. Bait proteins are indicated by asterisks. For semi-quantitative mass spectrometry analysis, see Table S2.

(B) Sucrose gradient analysis of Nop4-FTpA. FLAG eluate of affinity-purified Nop4 (input) was fractionated by sucrose gradient centrifugation (10%–40% sucrose) (w/v). Fourteen fractions were collected and proteins were precipitated by trichloroacetic acid (TCA). The resuspended fractions were analyzed by 4%–12% gradient SDS-PAGE and stained with Coomassie. Fraction #10 is depicted on the right and the associated factors were identified by mass spectrometry. Rrp5 module factors are colored in red and Urb1 module factors in orange. The Nop4 bait protein is indicated by an asterisk.

essential role in the Rrp5-Noc1-Noc2 module that is part of the early pre-60S, with Rrp5 potentially bridging to the 90S (see section "introduction"). Urb2 (also named Npa2) was chosen because it is part of a chaperone complex, Urb1-Urb2-Nop8-Rsa3-Dbp6, that was also found in association with early pre-60S particles, 90S factors, and several snoRNAs (Dez et al., 2004; Joret et al., 2018; Rosado et al., 2007). As anticipated, Urb2-Noc1 purification yielded a unique pre-60S pattern of bands with stoichiometrically co-enriched Rrp5 and Urb1 modules, as well as a number of typical pre-60S factors (e.g., Erb1, Nop7, Nsa3) (Figure 2A; Table S2), but also poorly investigated pre-60S factors (e.g., Nop4, Figure 2B; see also below). To our surprise, we also found enrichment of the C/D box snoRNA core factors (e.g., Nop1, Nop58, and Nop56 were all visible as Coomassie-stained bands), but other 90S factors were mostly absent. When other split-tag combinations were tested with Urb2 as first bait, but Nsa3 (a pre-60S foot factor) or Nop58 (a C/D box snoRNA core factor) as second baits, we noticed a similar pattern of co-enriched bands to that observed for the Urb2-Noc1 preparation (Figure 2A).

The finding that the C/D box snoRNA core factors are prominent constituents of the primordial pre-60S encouraged us to identify the associated snoRNAs. We analyzed the co-precipitated RNAs in these various preparations by polyacrylamide/urea gel electrophoresis and SYBR Green staining. This revealed two major RNA bands, approximately 200 and 400 nucleotides in length, that were strongly enriched in Urb2-Noc1 and Urb2-Nop58 particles, but not detected in the nuclear pre-60S (Arx1) and 90S (Noc4-Dhr1) particles (Figure 3A). Based on their sizes, we tentatively assigned these RNAs to the C/D box snR190 and the H/ACA box snR37 (Figure 3A and S3A), which was subsequently confirmed by northern analysis (Figure 3B) and affinity purification of the Urb2-Noc1 particle from an *snR190Δ snR37Δ* double-knockout strain (see below), which specifically no longer revealed these two snoRNA bands (Figure S2). To demonstrate that snR190 and snR37 are stably associated with the earliest pre-60S particles, we fractionated the Urb2-Nop58 eluate by sucrose gradient centrifugation. Both snR190 and snR37 co-migrated with the pre-60S particles in the lower part of the sucrose gradient (Figure 3C).

To identify additional snoRNAs of lower abundance in the early pre-60S particles, we performed small RNA Illumina sequencing of the total RNA extracted from the different preparations, that is, from the Urb2-Noc1 and Urb2-Nop58 eluates, from a Nop58 affinity purification to detect the entire pool of C/D box snoRNAs, from Kre33 as bait for 90S, and from Arx1 as bait for nuclear and cytoplasmic pre-60S particles (Figure S3B; Table S3).

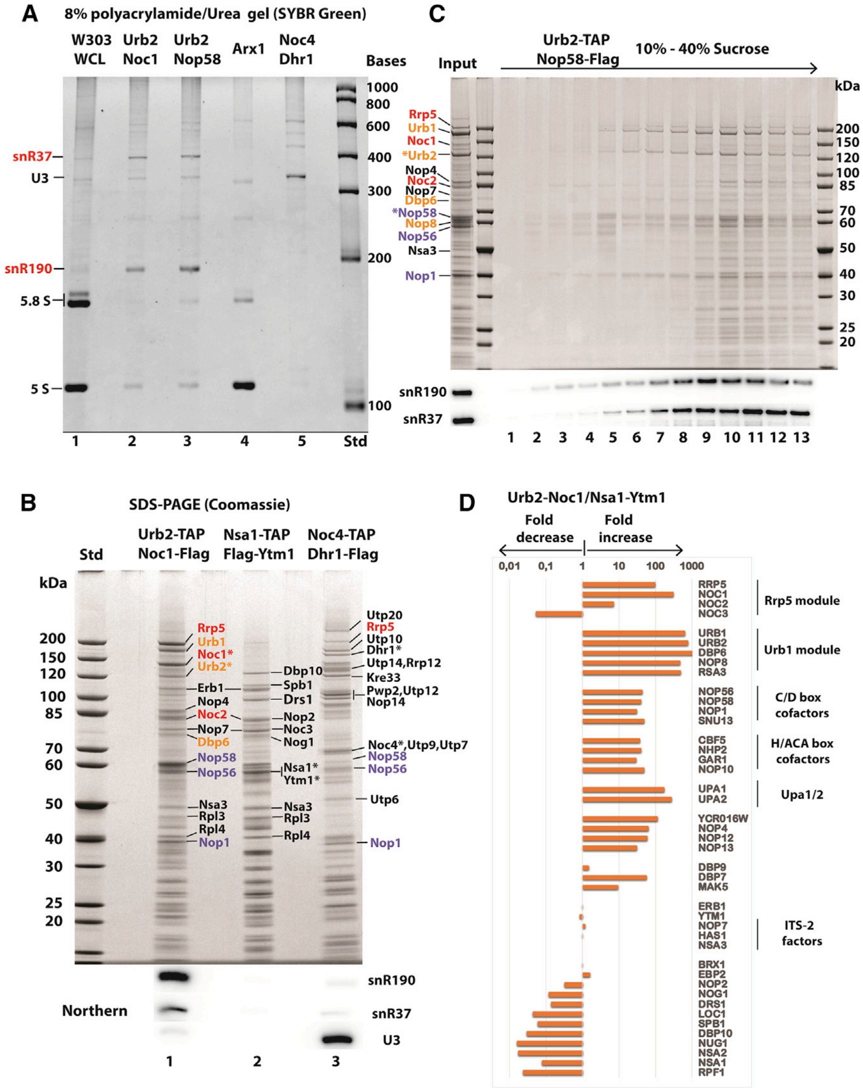

Figure 3. Northern analysis and SYBR Green RNA staining of pre-60S and 90S particles

(A) Low-molecular-weight RNA from the split-tag affinity-purified Urb2-Noc1, Urb2-Nop58, Arx1, and Noc4-Dhr1 pre-ribosomal particles was analyzed by 8% polyacrylamide/urea gel electrophoresis. Total RNA from a wild-type strain W303 whole-cell lysate (WCL) and the final eluates from the purifications were separated on the gel and stained for RNA with SYBR Green.

(B) Split-tag affinity purification of Urb2-Noc1, Nsa1-Ytm1, and Noc4-Dhr1 particles. The final eluates were divided: one-half was analyzed by 4%–12% gradient SDS-PAGE and Coomassie staining (top) to reveal the protein composition; the other half was used to extract RNA, which was analyzed by northern blotting using specific probes for the snoRNAs snR190, snR37, and U3 (bottom).

(C) FLAG eluate of affinity-purified Urb2-TAP-Nop58-FLAG particle was fractionated by sucrose gradient centrifugation (10%–40% sucrose gradient). A portion of each fraction was used for RNA extraction and northern blotting using probes for snR190 and snR37, and the remainder was loaded onto a 4%–12% gradient SDS-PAGE gel and stained with Coomassie. Rrp5 module factors are colored in red, Urb1 module factors in orange, and C/D box snoRNA cofactors in blue. Bait proteins are indicated by asterisks.

(D) Semi-quantitative mass spectrometry analysis of the final Urb2-Noc1 and Nsa1-Ytm1 eluates. The label-free quantification values (normalized to Nsa3) of the co-enriched assembly factors are compared by dividing the values from the Urb2-Noc1 preparation by those from the Nsa1-Ytm1, indicating the fold decrease or increase. For the whole dataset of the mass spectrometry analysis, see Table S2.

Analysis of Illumina sequencing showed that Urb2-Noc1 and Urb2-Nop58 particles contained a similar set of snoRNAs to other preparations, with the highest enrichment for C/D box members snR190, snR73, snR38, snR39, snR48, and snR52, and H/ACA members snR37 and snR42 (Figure S3B). As anticipated, snR190 is clearly a major snoRNA in the Urb2-derived particles, but snR37 was less prominent (Table S3). The latter observation might be due to a technical limitation of the Illumina sequencing method, which is optimized for transcripts smaller than 200 nucleotides; by contrast, snR37 is an extraordinarily long (386 nucleotides) snoRNA.

To systematically delineate the primordial from the succeeding classical pre-60S particles, we compared the ratios of assembly factor increase or decrease by semi-quantitative mass spectrometry (Figures 3D; Table S2; see also Figure 3B). This confirmed that the Rrp5 module (Rrp5-Noc1-Noc2), Urb1 module (Urb1-Urb2-Dbp6-Nop8-Rsa3), C/D box (Nop58-Nop56-Nop1-Snu13), and H/ACA snoRNA core (Cbf5-Nhp2-Gar1-Nop10) factors were strongly enriched in the Urb2-Noc1 preparation compared with the well-described Nsa1-Ytm1 nucleolar pre-60S particles (Kater et al., 2017) (Figure 3D). The Urb2-Noc1 particles also contained a number of poorly characterized RNA helicases, such as Dbp6, Dbp7, Dbp9, and Mak5, of which only Dbp9 is retained in the later Nsa1-Ytm1 particles (Figure 3D). These RNA helicases might assist early pre-rRNA folding or remove snoRNAs and assembly factors from the developing pre-60S. Another characteristic component of the primordial pre-60S was Nop4/Nop77 (see also Figure 2B), an essential but little-studied biogenesis factor of the large subunit (Sun and Woolford, 1994), which was initially found in a synthetic lethality screen with another *nop1* mutant allele, *nop1-5* (Bergès et al., 1994). Previously, Nop4 was not recovered in the classical pre-60S particles (Kater et al., 2017), consistent with its participation in the earliest endonucleolytic cleavages within ITS1 and its cross-linkable position near the 5′ end of 5.8S rRNA (Granneman et al., 2011). Mutations in the human ortholog of Nop4, RBM28, contribute to acute necrotizing encephalopathy syndrome, which is a

ribosomopathic-like disease (Nousbeck et al., 2008). To demonstrate that Nop4 is a specific factor of the primordial pre-60S, we affinity purified Nop4 and analyzed it by sucrose gradient centrifugation. This revealed association of Nop4 with early pre-60S factors that were mainly detected in fraction 10 of the sucrose gradient (e.g., Rrp5, Noc1, Noc2, Urb1, Urb2), but another pool of Nop4 also sedimented in other parts of the gradient (Figure 2B). Further unique factors in the Urb2-Noc1 particle are an uncharacterized protein YCR016W (a conserved RNA-binding protein; see section "discussion"), Nop12-Nop13, which together with Pwp1 participate in early 5.8S rRNA folding (Talkish et al., 2014), and a putative methyltransferase YGR283C (termed Urb2-particle-associated methyltransferase 1 [Upa1]) and its paralog YMR310C (accordingly termed Upa2), the latter two being approximately 200–300-fold enriched (Figure 3D; for further characterization, see below).

By contrast, a series of other pre-60S factors with assigned functions and/or distinct positions in the structurally analyzed nucleolar pre-60S particles (e.g., Nog1, Drs1, Dbp10, Spb1, Loc1, Nug1, Nsa2, Nsa1, and Rpf1) were markedly diminished in the Urb2-Noc1 particles (Figure 3D; Table S2). Notably, the band corresponding to the ribosomal protein Rpl3 in the SDS-polyacrylamide gel of the Urb2-Noc1 eluate was clearly less intense than the Rpl4 band, but both have comparable intensities in the classical pre-60S particles (Figures 2A and 3B). This observation might suggest that Rpl3 awaits its incorporation into the primordial pre-60S, whereas Rpl4 was already recruited (see section, "discussion"). We also noticed a few sub-stoichiometric 90S factors in the Urb2-Noc1 preparation, likely from a pool of particles that did not undergo endonucleolytic ITS1 cleavage and hence 90S and pre-60S moieties were not yet separated (see below).

Considering all these findings, the nascent 60S subunit that precedes the previously described nucleolar pre-60S particle is characterized by, among other features, the presence of two large α-solenoid scaffold complexes (Urb1 and Rrp5 modules), a cluster of little-studied RNA helicases, and two prominent snoRNPs (snR190 and snR37).

Role of snR190 and snR37 in the primordial pre-60S particles

To investigate the roles of snR190 and snR37 that are so prominent in the earliest pre-60S particles, we conducted genetic and biochemical studies. It is known that snR190 does not guide 2′-*O*-ribose methylation in the ribosomal RNA (Kiss-László et al., 1996; Taoka et al., 2016; Yang et al., 2016), and hence might have a structural role in the earliest pre-60S intermediates, as does the U3 snoRNA in the 90S pre-ribosomes. By contrast, the H/ACA snR37 is computationally predicted to function as a guide snoRNA for pseudouridylation at position U2944 in yeast 25S rRNA (Piekna-Przybylska et al., 2007; Schattner et al., 2004); however, experimental evidence for this is lacking. As previously shown and confirmed here, neither snR190 nor snR37 are essential for cell growth in yeast (Figure 4A) (Balakin et al., 1996; Zagorski et al., 1988), but an isogenic *snR190Δ snR37Δ* double-disruption strain, generated in this study, induced a synergistic growth defect at 37°C (Figure 4A). To determine whether this was caused by an assembly defect of the primordial pre-60S, we affinity purified Urb2-Noc1 from the single-disruption strains, as well as from the *snR190Δ snR37Δ* double mutant, which was devoid of these two snoRNAs (Figure S2). To reveal any changes in the pattern of co-enriched assembly factors, we performed SDS-PAGE and Coomassie staining, and compared the different preparations by semi-quantitative mass spectrometry (Figures 4B and 4C; Table S4). For the single *snR190Δ* deletion, we observed a specific reduction of the RNA helicases Dbp7, Dbp9, and Mak5, whereas the bulk of the other factors was unaffected (Figures 4B and 4C). In addition, the Ssf1/Ssf2-Rrp15-Mak11 subcomplex was diminished, which nevertheless was only sub-stoichiometrically detected in the wild-type Urb2-Noc1 particles (Figures 4C and Table S4). The most striking difference, however, was the complete absence of the two putative methyltransferases Upa1 and Upa2 from the Urb2-Noc1 particle when isolated from the *snR37Δ* strain, but not when purified from the *snR190Δ* strain (Figures 4C and Table S4). Concomitantly, we observed a reduction of H/ACA core factors (e.g., Cbf5 and Nhp2) in the Urb2-Noc1 particles when affinity purified from the *snR37Δ* strain. All these alterations detected in the single-deletion mutants were sustained in the *snR190Δ snR37Δ* double-deletion strain, except for YCR016W, which showed a synergistically enhanced reduction. Thus, YCR016W, which encodes a conserved putative RNA-binding protein (human homolog C7orf50), might contribute to the synthetically enhanced phenotype observed between snR190 and snR37.

To find evidence that snR190 is genetically linked to biogenesis factors of the primordial pre-60S, we combined *snoR190Δ* with the *rsa3Δ* gene disruption, which yielded a synthetically enhanced (*se*) growth defect at all temperatures tested (Figure S4A). Rsa3 is a nonessential component of the Urb1 module (Rosado et al., 2007) that is reported to associate with different snoRNAs, including snR190 (Joret et al., 2018). Subsequent isolation of Nsa3-derived pre-60S particles from the *snoR190Δ rsa3Δ* double mutant revealed an altered pattern of co-purifying factors, with accumulation of 90S factors (e.g., Utp20, Utp22, Rrp7, Pwp2, Utp10, Rrp12, Kre33), reminiscent of what has been found for Nsa3 particles isolated from the *nop1-4* strain. However, a specific set of other pre-60S factors, including Dbp10, Spb1, and Nug1, were decreased if Nsa3 was purified from the *snoR190Δ rsa3Δ* double mutant (Figure S4B and S4C; Table S5).

SPOUT methyltransferases Upa1 and Upa2 are suitable single baits that enrich the primordial pre-60S

Since Upa1 and Upa2 were found to be specific factors of the Urb2-Noc1 particle with a link to snR37, we further analyzed these uncharacterized paralogs. Both belong to a group of methyltransferases that carry the SPOUT domain, with representatives in all three domains of life. SPOUT-class proteins can bind *S*-adenosyl-L-methionine and facilitate the transfer of a methyl group to the substrate, which can be RNA or protein (Anantharam et al., 2002; Tkaczuk et al., 2007). The yeast paralogs Upa1 and Upa2 were predicted to methylate RNA, but no such modification of rRNA has been demonstrated (Szczepińska et al., 2014). Hence, we affinity purified

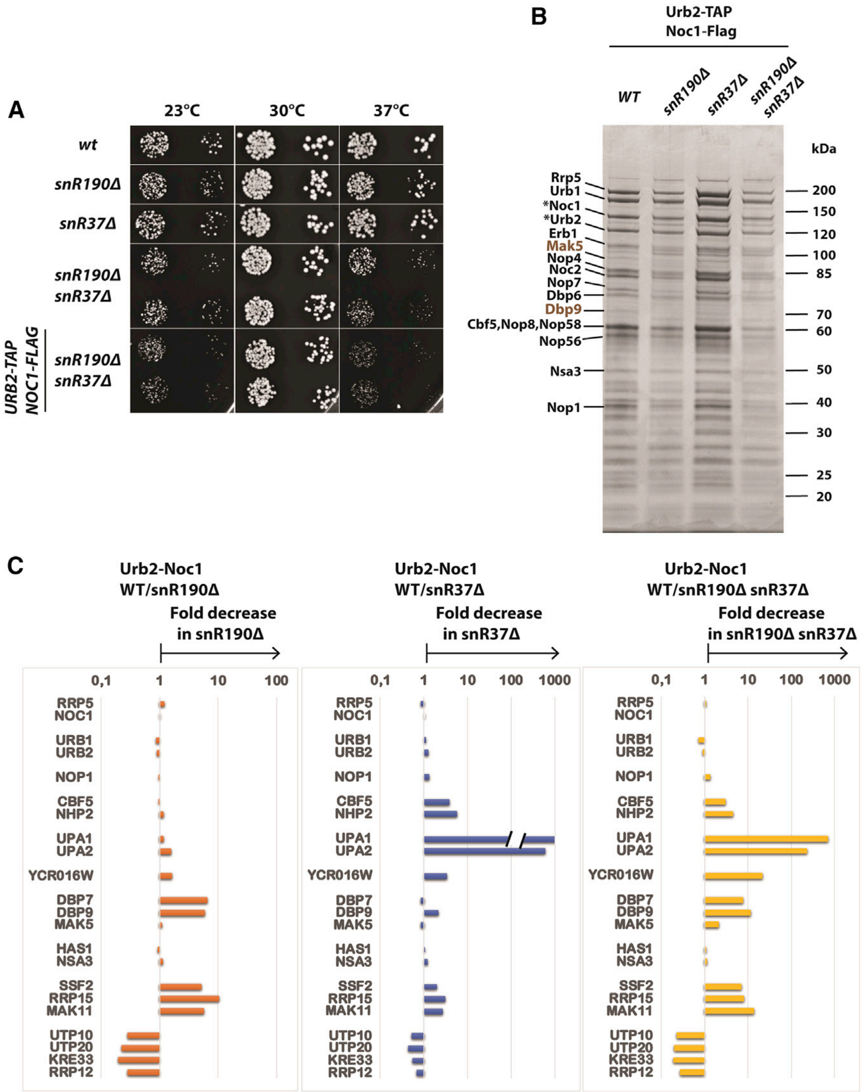

Figure 4. Deletion of snR190 and snR37 causes specific changes in the Urb2-Noc1 particle

(A) To compare their growth, the W303 (isogenic wild type), *snR190Δ* and *snR37Δ* single-deletion, and *snR190Δ snR37Δ* double-deletion strains were spotted in 10-fold serial dilutions on YPD plates. Plates were incubated at 23°C, 30°C, or 37°C for 2 days.

(B) Affinity purification of Urb2-TAP-Noc1-FLAG from the wild-type, *snR190Δ* and *snR37Δ* single-deletion, and *snR190Δ snR37Δ* double-deletion strains. FLAG eluates were analyzed on a 4%–12% gradient SDS-PAGE gel and stained with Coomassie. Mak5 and Dbp9 are labeled in green to show their reduction in the *snR190Δ* strain.

(C) Semi-quantitative mass spectrometry analysis of the final FLAG eluates from the strains described for (B). The label-free quantification (LFQ) values were normalized to Noc1 in each sample. Normalized LFQ values from the wild-type were divided by those of the individual snoRNA deletion strains (snR190Δ, snR37Δ, and snR190Δ snR37Δ); these ratios are represented on a bar graph to show the fold reduction of each factor in the mutant. Note that Upa1 was not detected in the snR37Δ strain (LFQ value of 0). In this case, we arbitrarily set a low LFQ value of 1 for the calculation. WT, wild type. For semi-quantitative mass spectrometry analysis, see Table S4.

FTpA-tagged Upa1 and Upa2 from yeast, which yielded an almost identical pattern of co-enriched bands for both baits. This is highly similar to what we observed only with the split-bait combination Urb2-Noc1. Interestingly, the Upa1 or Upa2 particles carry, among other factors, the Rrp5 and Urb1 modules, H/ACA and C/D box core factors, Nop4, Nop12, Nop13, YCR016W, and the RNA helicases Dbp7, Dbp9, and Mak5 (Figure 5; Table S6). Apparently, the Upa1 preparation contained Upa2 and vice versa, verified by either MALDI-TOF of the corresponding gel-excised bands (Figure 5A) or semi-quantitative mass spectrometry (Figure 5B; Table S6). Accordingly, the two paralogs Upa1 and Upa2, which arose in *S. cerevisiae* from an ancient whole-genome duplication, might form a dimer; this is similar to the human SPOUT ortholog C9orf114 (SPOUT1) and other proteins from this family, including Emg1, that function as homodimers (see section, "discussion"). In the past, affinity purification of early pre-60S factors such as Rrp5, Noc1, Urb1, Nop8, and Rsa3 revealed co-enrichment of the $27SA_2$ pre-rRNA (Dez et al., 2004; Hierlmeier et al., 2013; Jaafar et al., 2021; Joret et al., 2018). When we probed the Urb2-Noc1 and Upa1 preparations by northern, we also detected the $27SA_2$ pre-rRNA (data not shown).

To gain a first insight into their structure, we analyzed the Upa1-enriched pre-60S particles by negative-stain EM and 2D classification. According to the results, Upa1 particles are approximately 32 nm long and 23 nm wide, and are typically U shaped in appearance (Figure 5C). In a number of 2D classes, a blurry extra density at one tip of the U could be discerned, which might correspond to a flexibly attached substructure, for which Rrp5, Urb1, and snoRNP modules are good candidates. This early pre-60S is highly flexible and awaits the stable integration and compaction of the subdomains and modules. This tendency is also apparent in the case of some of the succeeding and structurally better characterized pre-60S particles (e.g., state A or B pre-60S particles in Kater et al., 2017; state 1, 2, and 3 pre-60S particles in Sanghai et al., 2018), which still contain a few flexible modules and/or subdomains (e.g., 5S RNP, Ssf1 module, domains III, IV, V, and VI).

Mak5 helicase mutant allows structural analysis of a bipartite 90S-pre-60S particle

Mak5—known to play a role in early pre-60S assembly (Bernstein et al., 2006; Brüning et al., 2018; Pratte et al., 2013)—is

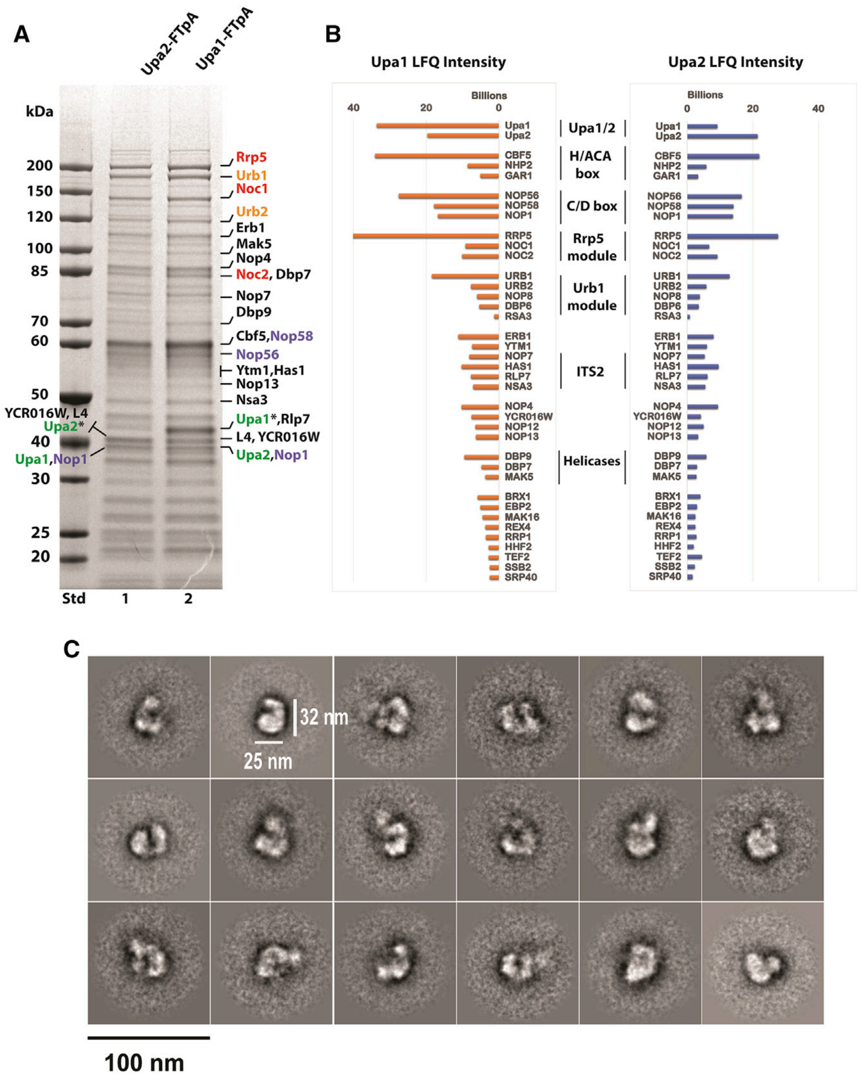

Figure 5. The methyltransferases Upa1 and Upa2 enrich early pre-60S particles upon their affinity purification similar to the pattern of Urb2-Noc1 particles

(A) Affinity purification of Upa1-FTpA and Upa2-FTpA. FLAG eluates were analyzed on a 4%–12% gradient SDS-PAGE gel and stained with Coomassie. The major labeled bands, including Upa1 and Upa2, were excised from the gel and identified by mass spectrometry. The Rrp5 module is labeled in red, Urb1 module in orange, C/D box snoRNA cofactors in blue, and Upa1/Upa2 in green. Bait proteins are indicated by asterisks.

(B) Semi-quantitative mass spectrometry of the eluates from Upa1 and Upa2 purifications. The absolute LFQ intensities are represented in the bar graph and the assembly factors are categorized. For the whole dataset of the mass spectrometry analysis, see Table S6.

(C) Negative-stain EM of Upa1-FTpA preparation. Typical 2D classes of these early pre-60S particles are shown. Scale bar: 100 nm.

specific but somewhat sub-stoichiometric in the Urb2-derived pre-60S particles (Figure 2A). Association of Mak5 with Urb2-Noc1 particle appeared affected upon snR190 deletion (Figure 4B). Therefore, we wanted to further analyze this essential RNA helicase, for which a dominant-negative mutant mapping in the Walker B motif (Mak5 D333A, defective in ATP hydrolysis) has been described (Bernstein et al., 2006). We expressed *mak5* D333A under the control of the *GAL* promoter in the yeast strains expressing different baits for isolation of the various pre-60S particles (for the dominant-negative phenotype, see Figure S5A). Evidently, the dominant Mak5 D333A band was further enriched in the Urb2-Noc1 particles compared with the wild-type Mak5, but many 90S factors also accumulated (e.g., Utp20, Utp10, Utp22, Bms1, Rrp12, Utp21, Pwp2), whereas ribosomal Rpl3 was distinctly absent (Figure 6A). Similarly, but much more pronounced, dominant Mak5 D333A was strongly trapped on the later Nsa3 and Nsa1-Ytm1 pre-60S particles, accompanied by clear decreases in the methyltransferase Spb1, the GTPase Nug1, and the export factor Arx1 (Figure 6B). Interestingly, the Nug1 N terminus and Spb1 C terminus, which are present in the cryo-EM structure of state D Nsa1-Ytm1 pre-60S particles (Kater et al., 2017), are close to the 25S rRNA CRAC (cross-linking and analysis of cDNAs) crosslink sites of Mak5 (Brüning et al., 2018) (Figure S5B). This correlation suggests that Mak5 D333A, unable to hydrolyze ATP, might not efficiently dissociate from its binding site, thereby blocking successive recruitment of Spb1 and Nug1 to this position on the pre-60S.

Based on these biochemical data, the Urb2-Noc1 particles isolated from the Mak5 D333A dominant mutant appeared appropriate for structural analysis. We performed negative-stain EM first, which revealed smaller-sized particles (~32 nm in diameter), medium-sized particles (~42 nm), and very large bipartite particles (~70 nm in length, 40 nm in width); the latter far exceed the size of either pre-60S or 90S particles (Figure 6C). Accordingly, the smaller particles might correspond to the primordial pre-60S, as was found for the Upa1 preparation (see also Figure 5C). The medium-sized particles could be classical 90S, which indeed greatly resemble previously studied 90S particles from yeast and *Chaetomium thermophilum*, and the particles of approximately 70 nm might be a bipartite assembly between 90S and pre-60S particles, consistent with the biochemical data. In accordance with this interpretation, the larger of the two halves of the bipartite particle exhibits the typical 90S structure as seen by negative-stain EM, whereas the smaller half appears less distinct and more flexible, but resembles in its overall shape the primordial pre-60S (Figure 6C). Thus, the bipartite large-sized particle could be the long-sought 90S-pre-60S pre-ribosome.

To gain deeper insight into the structure of these different particles, we performed cryo-EM of the Urb2-Noc1-Mak5-333

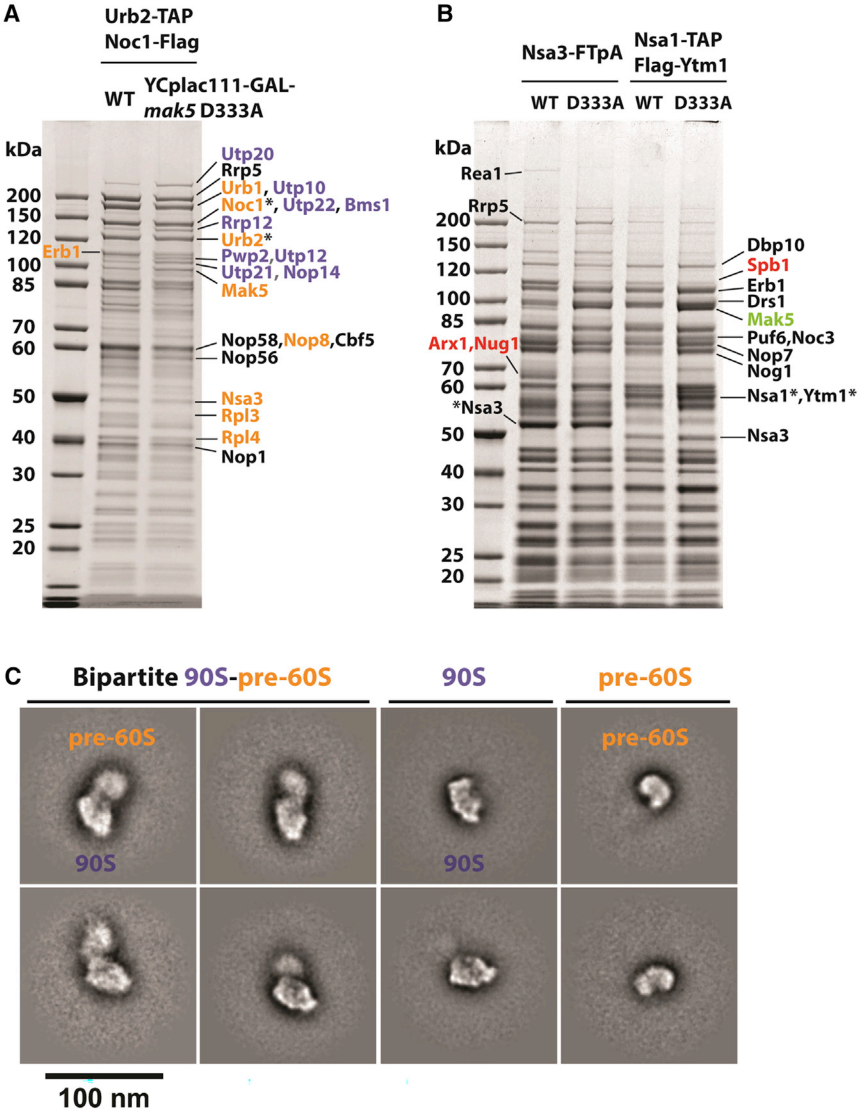

Figure 6. Isolation of bipartite 90S-pre-60S particles from the dominant Mak5 D333A mutant

(A) Isolation of Urb2-TAP-Noc1-FLAG particle (WT) and after overexpression of Mak5 D333A. The Urb2-TAP Noc1-FLAG strain was transformed with YCplac111-GAL-*mak5* D333A. Cultures were grown in glucose then shifted to galactose and grown for 8 h. FLAG eluates were analyzed on a 4%–12% gradient SDS-PAGE gel and stained with Coomassie. Major bands were identified by mass spectrometry. 90S assembly factors are colored in blue and pre-60S factors are colored in orange. Bait proteins are indicated by asterisks.

(B) Isolation of Nsa3-FTpA and Nsa1-TAP FLAG-Ytm1 particles with overexpressed wild-type Mak5 and Mak5 D333A mutant. The Nsa3-FTpA and Nsa1-TAP FLAG-Ytm1 strains were transformed with YCplac111-GAL-*MAK5* or *mak5* D333A. Cultures were grown in glucose then shifted to galactose and grown for 8 h. FLAG eluates were analyzed on a 4%–12% gradient SDS-PAGE gel and stained with Coomassie. Major bands were identified by mass spectrometry. The assembly factors Spb1, Nug1, and Arx1 are colored in red to highlight their reduction in the case of Mak5 D333A overexpression. The overexpressed Mak5 is colored in light green. Bait proteins are indicated by asterisks. WT, wild type.

(C) Negative-stain EM of the Urb2-TAP Noc1-FLAG YCplac111-GAL-*mak5* D333A sample. Two-dimensional classes showing bipartite 90S-pre-60S particles with 90S attached to a flexible fuzzy pre-60S mass. Individual 90S and pre-60S particles from the same sample are also shown for comparison. Scale bar: 100 nm.

preparation, from which we obtained cryo-EM maps for the major intermediates, albeit only at lower resolution, but sufficient for a prudent assignment (Figure 7). The smaller particles greatly resemble the early pre-60S particles of the Nsa1-Nop2 state 3 (Sanghai et al., 2018), exhibiting a distinct ITS2 foot structure, domain I–II-5.8S rRNA, and the Erb1-Ytm1 module, but flexible domains IV, V, and VI (Figure 7A). In our case, the large modules such as the Rrp5-Noc1-Noc2 or the Urb1 complex are also not visible, most likely also due to their flexible attachment. The medium-sized particles are similar to classical 90S particles (e.g., Noc4-Dhr1 pre-A_1. EMDB: 11359; Cheng et al., 2020), with typical hallmark structures and module factors such as Kre33, Rrp7, Utp22, Utp13, Utp12, and h44 of the 18S rRNA (Figure 7B). Re-extraction of the 90S particles with a larger box size led to the appearance of extra density near the head of the 90S, which resembled in dimension early pre-60S particles. This extra density, however, could not be further resolved, indicating a high degree of expected flexibility and conformational plasticity (Figure 7C). Based on its overall dimension, our biochemical data, and the negative stain results, we can ascribe this additional but blurry density to the pre-60S. It is directly attached to the 90S lobe, emerging from the area where the Utp22-Rrp7 module of the 90S is located (Figure 7C). This finding suggests that physical contact between the pre-60S and the 90S in our bipartite particle occurs in the region of the UTP-C module, where Rrp5 is also located. This interpretation is consistent with Rrp5 being a bridging factor between the small and large pre-ribosome, with major contact points at the bridging ITS1 rRNA (see section "introduction"; Lebaron et al., 2013). However, Rrp5 is not well resolved in the 90S moiety of our bipartite 90S-pre-60S particle, but part of it (C-terminal residues 1,457–1,721) is clearly seen in the Noc4-Dhr1 90S particle, in which this region of Rrp5 is in close contact with Utp22. Considering these collective structural findings, we can derive a first visual understanding of how the nascent small and large subunits are joined at the early stage of eukaryotic ribosome assembly, before the two maturation pathways diverge. Presumably, due to the highly unstructured nature of the very early

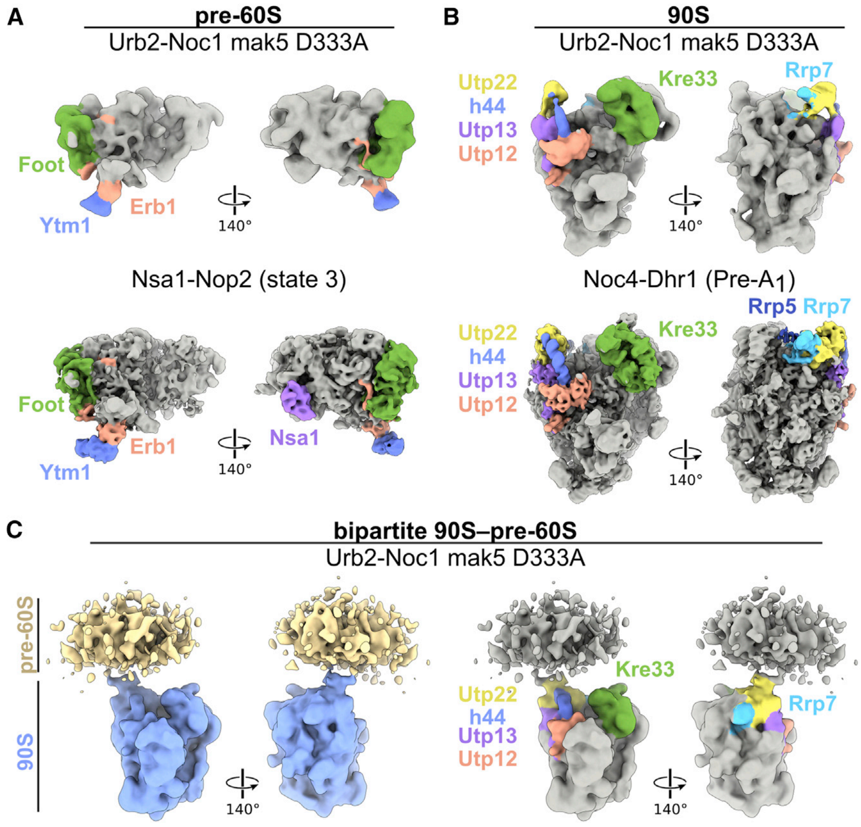

Figure 7. Cryo-EM structure of the bipartite 90S-pre-60S particle

(A) Comparison of the Urb2-Noc1 mak5 D333A pre-60S cryo-EM reconstitution (top) with the Nsa1-Nop2 map (state 3, EMDB: 7445. PDB: 6CB1; bottom) lowpass-filtered to 10 Å. The foot structure (consisting of Nsa3, Nop7, Rlp7, and Nop15), Erb1, Ytm1, and Nsa1 are labeled.

(B) Comparison of the obtained Urb2-Noc1 mak5 D333A 90S cryo-EM map (top) with the Noc4-Dhr1 pre-A_1 particle-filtered to 10 Å (EMDB: 11359. PDB: 6ZQC; bottom). Utp12, Utp13, Utp22, Kre33, Rrp7, and Rrp5 are highlighted.

(C) Gaussian filtered cryo-EM map of the 90S particle connected to a flexible extra density likely containing the emerging pre-60S subunit. (Left) The 90S and the extra density colored in blue and yellow, respectively. (Right) The cryo-EM map in gray and Utp12, Utp13, Utp22, Kre33, and Rrp7 are highlighted. For the sorting scheme of the particles, see Figure S6. For the structure prediction of Urb1 and Rrp5 module members, which are not visible on the pre-60S particles due to flexibility, see Figure S7.

nucleolar pre-60S subunit, a better resolution appeared to be impossible with the given sample.

DISCUSSION

The results of this study give us a molecular insight into the early phase of the pathway eukaryotes use to assemble the large ribosomal subunit. This revealed a primordial pre-60S particle that is dominated by two scaffold modules, Rrp5-Noc1-Noc2 and Urb1-Urb2-Nop10-Dbp6-Rsa3, and further distinguished by two prominent snoRNPs, snR190 and snR37; several poorly characterized RNA helicases, including Mak5; and the SPOUT-domain-containing methyltransferase pair Upa1-Upa2. The huge and α-solenoid-dominated Urb1-and Rrp5-containing modules (Figure S7) and the snoRNPs could significantly contribute to the pre-large-subunit knobs initially visualized by the Miller spread technique on chromatin spreads, which appear after co-transcriptional cleavage of the nascent 35S pre-rRNA in the ITS1 region (Osheim et al., 2004). Similar pre-small-subunit terminal knobs have also been attributed to large α-solenoid proteins (e.g., members of UTP-A and UTP-B, Utp20) and the U3 snoRNP (Miller and Beatty, 1969; Mougey et al., 1993; Osheim et al., 2004). Importantly, by disrupting the ribosome assembly pathway upon induction of a dominant-negative *mak5* helicase mutant, we could arrest the early pre-60S maturation pathway at a stage at which the newly forming large subunit is still connected to the enormous precursor of the 40S, the 90S pre-ribosome. This welcome side effect allowed us to reveal for the first time the overall shape of the largest assembly intermediate in the eukaryotic ribosome biogenesis pathway: the bipartite 90S-pre-60S particle.

The two principal snoRNAs in the primordial pre-60S, snR190 and snR37, which are C/D box and H/ACA snoRNAs, respectively, are not shown to be essential when individually deleted, but the snR190 snR37 double disruption induced a synergistic growth defect at higher temperatures. Notably, the uncharacterized protein YCR016W, which exhibits a unique WKF domain (previously called DUF2373 domain), like its human ortholog C7orf50, which has been shown to bind to RNA (Trendel et al., 2019), was synergistically reduced in the primordial pre-60S particle. In genome-wide screens in yeast, both negative and positive genetic interactions between YCR016W and factors of the primordial pre-60S (e.g., Dbp6, Rsa3, Mak11, Drs1, Cbf5) were found (Costanzo et al., 2016), suggesting that association of YCR016W with the Urb2-Noc1 particle might involve direct snoRNA binding, explaining why YCR016W association was reduced by the snR190 snR37 double disruption. Thus, YCR016W could coordinate, due to its RNA-binding capability, the interactions between different snoRNAs on the earliest pre-60S particle.

Because snR190 does not guide a known base modification, it might instead play a structural role in the primordial pre-60S, as U3 snoRNP does in the 90S pre-ribosome. Specifically, snR190 provides a linkage to the Urb1 module, with direct contact to Urb1, demonstrable by CRAC analysis (Joret et al., 2018) and, as found here, a genetic link to the Urb1-complex factor Rsa3. One scenario to explain these data could be that snR190 binds to the nascent 27S pre-rRNA in a critical region to keep it protected from premature folding and/or engagement with other

CellPress
OPEN ACCESS

pre-60S factors that only later come into play. At this stage, Rpl3 might not be able yet to incorporate into the primordial pre-60S, because factors such as the Urb1 module could occupy the Rpl3 binding region (Joret et al., 2018). In this way, the Urb1 module in combination with snR190 could hinder recruitment of the Ssf1 module with attached Rpl3 to the primordial pre-60S, and only at a later step, when these factors are released (Sanghai et al., 2018), can Rpl3 reach its mature position, coupled with bringing together the involved root helices (Gamalinda et al., 2014). A related structural role could also be considered for snR37, which has, nonetheless, been predicted to aid pseudouridylation of the nascent 25S rRNA (Piekna-Przybylska et al., 2007; Schattner et al., 2004). Thus, the snR37 snoRNP could first play a structural role in the early pre-60S pathway, before terminating this with a decisive pseudouridylation on the pre-rRNA, authorizing it to proceed in the pathway.

The pre-60S particles that were isolated via the Urb2-dependent split-tag approaches were significantly enriched in several snoRNAs and a cluster of RNA helicases. Three of these helicases, Dbp7, Dbp9, and Mak5, were reduced if the Urb2-Noc1 particle was isolated from the snR190-disruption strain, which might point to direct functional link in the primordial pre-60S. Indeed, recent studies suggested that Dbp7 is involved in the release of snR190 from pre-60S particles (Aquino et al., 2021; Jaafar et al., 2021). By contrast, deletion of the C/D box snR190 did not substantially affect the co-enrichment of C/D box snoRNA core factors (e.g., Nop1 or Nop56) in the primordial pre-60S. This can be explained by the fact that a number of C/D box snoRNAs are present in the Urb2-Noc1 particle. Interestingly in this context, the *nop1-4* allele, which is expected to impair all C/D box snoRNPs, generated a strong defect in the early pre-60S assembly pathway, suggesting that the C/D box snoRNAs work synergistically together in the primordial pre-60S. However, deletion of snR37 clearly reduced H/ACA core factors (e.g., Cbf5 pseudouridine synthase), which is consistent with the finding that H/ACA snoRNAs are less prominent than C/D box snoRNAs in the primordial pre-60S.

Affinity purification of single-bait factors Upa1 and Upa2 enriched primordial pre-60S particles like the Urb2-based split purifications, making them superior for isolating these earliest large-subunit assembly intermediates. So far, very little is known about how Upa1 and Upa2 perform their role in this segment of the large-subunit assembly pathway, but genome-wide screens reveal a genetic linkage to Urb2, Nop8, Dbp6, and Dbp9 (Costanzo et al., 2016). We assume that the Upa1-Upa2 paralogs, which evolved though yeast genome duplication, function together as a heterodimer, based on findings from the human ortholog C9orf114 (SPOUT1), which forms and functions as a homodimer (PDB: 4RG1), or the SPOUT-domain-containing methyltransferase Emg1, which is present as a homodimer on 90S particles (Chaker-Margot et al., 2017; Cheng et al., 2020; Kornprobst et al., 2016). As Upa1 and Upa2 do not co-enrich on primordial pre-60S if the H/ACA snR37 is deleted, and Upa1 and Upa2 strongly co-enrich Cbf5, H/ACA snoRNPs appear to be directly linked to Upa1-Upa2. Interestingly, it was shown that a few SPOUT-class methyltransferases methylate only pseudouridines, such as Emg1, which in the yeast 18S rRNA methylates U1191 only after its pseudouridylation (i.e., ψ1191) by snR35 (Meyer et al., 2011), or bacterial RlmH, which methylates ψ1915 in the 23S rRNA (Ero et al., 2008; Purta et al., 2008). Thus, we speculate that Upa1-Upa2 can recognize and methylate a specific pseudouridine in the primordial pre-60S, generated earlier by one of the co-enriched H/ACA snoRNAs.

A common theme in our findings is that disrupting ribosome assembly, either by the *nop1-4* mutation, deletion of snoRNAs, or overexpression of Mak5 D333A, caused significant trapping of 90S factors on the primordial pre-60S. It is possible that changing the mode of endonucleolytic cleavage in ITS1 (switching from co-transcriptional A_2 to post-transcriptional A_3 cleavage) might stabilize these highly dynamic 90S-pre-60S assembly intermediates, consistent with earlier findings that depletion of the Drs1 helicase caused an enrichment of 90S factors on Nop7- or Nog2-isolated pre-60S particles (Talkish et al., 2016). Our data suggest that the dominant-negative Mak5 D333A caused 90S factors to accumulate on primordial pre-60S particles because the 90S moiety could not efficiently detach. This scenario made it possible to visualize the long-sought 90S-pre-60S bipartite particle, revealing how the 90S and pre-60S moieties are connected in the UTP-C-Rrp5 region. Interestingly, the 90S structure could be well discerned in these huge particles, as it was already well developed, but the attached pre-60S moiety was largely in a rudimentary state in which most of the rRNA domains and bound modules that appeared are likely to be still very flexible, as they were not yet incorporated or compacted in this earliest pre-60S intermediate. We speculate that similar 90S-pre-60S bipartite particles also exist in higher eukaryotes (including human), for which post-transcriptional ribosome assembly seems to be the preferred pathway (Gamalinda et al., 2014). Now that we could reveal the overall shape and dimension of the bipartite 90S-pre-60S particle, it may also be easier to search for these characteristic assembly intermediates in the cellular context (e.g., in the nucleolus) by *in situ* cryoelectron tomography (for a recent publication, see Erdmann et al., 2021).

In conclusion, we obtained insights into the earliest steps of pre-60S assembly and progression, which involve a specific network of pre-60S assembly factors and snoRNAs, and to which the upstream 90S pathway is intimately coupled. Understanding the molecular details of this earliest phase of large-subunit synthesis in yeast might support studies in other organisms, including human, aimed at furthering our understanding of the link between post-transcriptional ribosome assembly and diseases such as cancers and ribosomopathies.

Limitations of the study

One limitation of our study is the use of a dominant *mak5* helicase mutant to identify the bipartite 90S-pre-60S particle. Although this mutant facilitated the isolation of such a ribosome assembly intermediate and its first cryo-EM analysis, it is currently unclear under which physiological conditions such a huge and flexible assembly exists in wild-type cells. A search for such a bipartite particle in intact cells could be one focus of future research not only in yeast but also in other organisms, including human.

STAR★METHODS

Detailed methods are provided in the online version of this paper and include the following:

- KEY RESOURCES TABLE
- RESOURCE AVAILABILITY
  - Lead contact
  - Materials availability
  - Data and code availability
- EXPERIMENTAL MODEL AND SUBJECT DETAILS
  - Yeast strains
  - Bacterial strains
- METHOD DETAILS
  - Yeast strains
  - Tandem affinity purification of pre-ribosomal particles
  - RNA extraction and analysis
  - Sucrose gradient centrifugation
  - Mass spectrometry
  - Electron microscopy and image processing
  - Illumina sequencing of RNA
- QUANTIFICATION AND STATISTICAL ANALYSIS

SUPPLEMENTAL INFORMATION

Supplemental information can be found online at https://doi.org/10.1016/j.celrep.2022.110640.

ACKNOWLEDGMENTS

This work was supported by grants from the German Research Council (GRK1721 to R.B., and HU363/15-2 to E.H.), and from the European Research Council (ADG 741781 GLOWSOME to E.H.).

AUTHOR CONTRIBUTIONS

S.I. and E.H. conceived the study. S.I. generated yeast strains, and isolated and characterized the pre-ribosomal particles by SYBR Green staining, northern blotting, and semi-quantitative mass spectrometry analysis. D.F. conducted the negative-stain EM analysis, prepared grids, and collected data for cryo-EM. M.T. processed the cryo-EM data and analyzed it with R.B. J.V.G.-F. and L.R. performed and analyzed Illumina sequencing. S.I. and E.H. wrote the manuscript. All authors commented on the manuscript.

DECLARATION OF INTERESTS

The authors declare no competing interests.

Received: October 11, 2021
Revised: February 1, 2022
Accepted: March 16, 2022
Published: April 5, 2022

SUPPORTING CITATIONS

The following reference appears in the supplemental information: Jumper et al. (2021).

REFERENCES

Allmang, C., Kufel, J., Chanfreau, G., Mitchell, P., Petfalski, E., and Tollervey, D. (1999). Functions of the exosome in rRNA, snoRNA and snRNA synthesis. EMBO J. *18*, 5399–5410.

Anantharam, V., Koonin, E.V., and Aravind, L. (2002). SPOUT: a class of methyltransferases that includes spoU and trmD RNA methylase superfamilies, and novel superfamilies of predicted prokaryotic RNA methylases. J. Mol. Microbiol. Biotechnol. *4*, 71–75.

Aquino, G.R.R., Hackert, P., Krogh, N., Pan, K.T., Jaafar, M., Henras, A.K., Nielsen, H., Urlaub, H., Bohnsack, K.E., and Bohnsack, M.T. (2021). The RNA helicase Dbp7 promotes domain V/VI compaction and stabilization of inter-domain interactions during early 60S assembly. Nat. Commun. *12*, 1–16.

Axt, K., French, S.L., Beyer, A.L., and Tollervey, D. (2014). Kinetic analysis demonstrates a requirement for the Rat1 exonuclease in cotranscriptional pre-rRNA cleavage. PLoS One *9*, e85703.

Balakin, A.G., Smith, L., and Fournier, M.J. (1996). The RNA world of the nucleolus: two major families of small RNAs defined by different box elements with related functions. Cell *86*, 823–834.

Barrio-Garcia, C., Thoms, M., Flemming, D., Kater, L., Berninghausen, O., Baßler, J., Beckmann, R., and Hurt, E. (2016). Architecture of the Rix1-Rea1 checkpoint machinery during pre-60S-ribosome remodeling. Nat. Struct. Mol. Biol. *23*, 37–44.

Baßler, J., and Hurt, E. (2019). Eukaryotic ribosome assembly. Annu. Rev. Biochem. *88*, 281–306.

Baßler, J., Kallas, M., Pertschy, B., Ulbrich, C., Thoms, M., and Hurt, E. (2010). The AAA-ATPase Rea1 drives removal of biogenesis factors during multiple stages of 60S ribosome assembly. Mol. Cell *38*, 712–721.

Baßler, J., Ahmed, Y.L., Kallas, M., Kornprobst, M., Calviño, F.R., Gnädig, M., Thoms, M., Stier, G., Ismail, S., Kharde, S., et al. (2017). Interaction network of the ribosome assembly machinery from a eukaryotic thermophile. Protein Sci. *26*, 327–342.

Bergès, T., Petfalski, E., Tollervey, D., and Hurt, E.C. (1994). Synthetic lethality with fibrillarin identifies NOP77p, a nucleolar protein required for pre-rRNA processing and modification. EMBO J. *13*, 3136–3148.

Bernstein, K.A., Granneman, S., Lee, A.V., Manickam, S., and Baserga, S.J. (2006). Comprehensive mutational analysis of yeast DEXD/H box RNA helicases involved in large ribosomal subunit biogenesis. Mol. Cell. Biol. *26*, 1195–1208.

Brüning, L., Hackert, P., Martin, R., Sloan, K.E., and Markus, T. (2018). RNA helicases mediate structural transitions and compositional changes in pre-ribosomal complexes. Nat. Commun. *9*, 5383.

Chaker-Margot, M., Hunziker, M., Barandun, J., Dill, B.D., and Klinge, S. (2015). Stage-specific assembly events of the 6-MDa small-subunit processome initiate eukaryotic ribosome biogenesis. Nat. Struct. Mol. Biol. *22*, 920–923.

Chaker-Margot, M., Barandun, J., Hunziker, M., and Klinge, S. (2017). Architecture of the yeast small subunit processome. Science *355*, eaal1880.

Chanfreau, G., Rotondo, G., Legrain, P., and Jacquier, A. (1998). Processing of a dicistronic small nucleolar RNA precursor by the RNA endonuclease Rnt1. EMBO J. *17*, 3726–3737.

Cheng, J., Baßler, J., Fischer, P., Lau, B., Kellner, N., Kunze, R., Griesel, S., Kallas, M., Berninghausen, O., Strauss, D., et al. (2019). Thermophile 90S pre-ribosome structures reveal the reverse order of Co-transcriptional 18S rRNA subdomain integration. Mol. Cell *75*, 1256–1269.e7.

Cheng, J., Lau, B., La Venuta, G., Ameismeier, M., Berninghausen, O., Hurt, E., and Beckmann, R. (2020). 90S pre-ribosome transformation into the primordial 40S subunit. Science *369*, 1470–1476.

Costanzo, M., VanderSluis, B., Koch, E.N., Baryshnikova, A., Pons, C., Tan, G., Wang, W., Usaj, M., Hanchard, J., Lee, S.D., et al. (2016). A global genetic interaction network maps a wiring diagram of cellular function. Science *353*, aaf1420.

Cox, J., and Mann, M. (2008). MaxQuant enables high peptide identification rates, individualized p.p.b.-range mass accuracies and proteome-wide protein quantification. Nat. Biotechnol. *26*, 1367–1372.

Danecek, P., Bonfield, J.K., Liddle, J., Marshall, J., Ohan, V., Pollard, M.O., Whitwham, A., Keane, T., McCarthy, S.A., Davies, R.M., et al. (2021). Twelve years of SAMtools and BCFtools. Gigascience *10*, 1–4.

CellPress
OPEN ACCESS

Dez, C., Froment, C., Noaillac-Depeyre, J., Monsarrat, B., Caizergues-Ferrer, M., and Henry, Y. (2004). Npa1p, a component of very early pre-60S ribosomal particles, associates with a subset of small nucleolar RNPs required for peptidyl transferase center modification. Mol. Cell. Biol. *24*, 6324–6337.

Dragon, F., Compagnone-Post, P.A., Mitchell, B.M., Porwancher, K.A., Wehner, K.A., Wormsley, S., Settlage, R.E., Shabanowitz, J., Osheim, Y., Beyer, A.L., et al. (2002). A large nucleolar U3 ribonucleoprotein required for 18S ribosomal RNA biogenesis. Nature *417*, 967–970.

Erdmann, P.S., Hou, Z., Klumpe, S., Khavnekar, S., Beck, F., Wilfling, F., Plitzko, J.M., and Baumeister, W. (2021). In situ cryo-electron tomography reveals gradient organization of ribosome biogenesis in intact nucleoli. Nat. Commun. *12*, 1–9.

Ero, R., Peil, L., Liiv, A., and Remme, J. (2008). Identification of pseudouridine methyltransferase in Escherichia coli. RNA *14*, 2223–2233.

Gamalinda, M., Ohmayer, U., Jakovljevic, J., Kumcuoglu, B., Woolford, J., Mbom, B., Lin, L., and Woolford, J.L. (2014). A hierarchical model for assembly of eukaryotic 60S ribosomal subunit domains. Genes Dev. *28*, 198–210.

Ganot, P., Bortolin, M.L., and Kiss, T. (1997). Site-specific pseudouridine formation in preribosomal RNA is guided by small nucleolar RNAs. Cell *89*, 799–809.

Gasse, L., Flemming, D., and Hurt, E. (2015). Coordinated ribosomal ITS2 RNA processing by the Las1 complex integrating endonuclease, polynucleotide kinase, and exonuclease activities. Mol. Cell *60*, 808–815.

Goddard, T.D., Huang, C.C., Meng, E.C., Pettersen, E.F., Couch, G.S., Morris, J.H., and Ferrin, T.E. (2018). UCSF ChimeraX: meeting modern challenges in visualization and analysis. Protein Sci. *27*, 14–25.

Grandi, P., Rybin, V., Baßler, J., Petfalski, E., Strauß, D., Marzioch, M., Schäfer, T., Kuster, B., Tschochner, H., Tollervey, D., et al. (2002). 90S pre-ribosomes include the 35S pre-rRNA, the U3 snoRNP, and 40S subunit processing factors but predominantly lack 60S synthesis factors. Mol. Cell *10*, 105–115.

Granneman, S., Petfalski, E., and Tollervey, D. (2011). A cluster of ribosome synthesis factors regulate pre-rRNA folding and 5.8S rRNA maturation by the Rat1 exonuclease. EMBO J. *30*, 4006–4019.

Hierlmeier, T., Merl, J., Sauert, M., Perez-Fernandez, J., Schultz, P., Bruckmann, A., Hamperl, S., Ohmayer, U., Rachel, R., Jacob, A., et al. (2013). Rrp5p, Noc1p and Noc2p form a protein module which is part of early large ribosomal subunit precursors in S. cerevisiae. Nucleic Acids Res. *41*, 1191–1210.

Hunziker, M., Barandun, J., Petfalski, E., Tan, D., Delan-Forino, C., Molloy, K.R., Kim, K.H., Dunn-Davies, H., Shi, Y., Chaker-Margot, M., et al. (2016). UtpA and UtpB chaperone nascent pre-ribosomal RNA and U3 snoRNA to initiate eukaryotic ribosome assembly. Nat. Commun. *7*, 1–10.

Hunziker, M., Barandun, J., Buzovetsky, O., Steckler, C., Molina, H., and Klinge, S. (2019). Conformational switches control early maturation of the eukaryotic small ribosomal subunit. Elife *8*, e45185.

Jaafar, M., Contreras, J., Dominique, C., Martín-Villanueva, S., Capeyrou, R., Vitali, P., Rodríguez-Galán, O., Velasco, C., Humbert, O., Watkins, N.J., et al. (2021). Association of snR190 snoRNA chaperone with early pre-60S particles is regulated by the RNA helicase Dbp7 in yeast. Nat. Commun. *12*, 1–17.

Janke, C., Magiera, M.M., Rathfelder, N., Taxis, C., Reber, S., Maekawa, H., Moreno-Borchart, A., Doenges, G., Schwob, E., Schiebel, E., et al. (2004). A versatile toolbox for PCR-based tagging of yeast genes: new fluorescent proteins, more markers and promoter substitution cassettes. Yeast *21*, 947–962.

Joret, C., Capeyrou, R., Belhabich-Baumas, K., Plisson-Chastang, C., Ghandour, R., Humbert, O., Fribourg, S., Leulliot, N., Lebaron, S., Henras, A.K., et al. (2018). The npa1p complex chaperones the assembly of the earliest eukaryotic large ribosomal subunit precursor. PLoS Genet *14*, e1007597.

Jumper, J., Evans, R., Pritzel, A., Green, T., Figurnov, M., Ronneberger, O., Tunyasuvunakool, K., Bates, R., Žídek, A., Potapenko, A., et al. (2021). Highly accurate protein structure prediction with AlphaFold. Nature *596*, 583–589.

Kater, L., Thoms, M., Barrio-Garcia, C., Cheng, J., Ismail, S., Ahmed, Y.L., Bange, G., Kressler, D., Berninghausen, O., Sinning, I., et al. (2017). Visualizing the assembly pathway of nucleolar pre-60S ribosomes. Cell *171*, 1599–1610.e13.

Khoshnevis, S., Liu, X., Dattolo, M.D., and Karbstein, K. (2019). Rrp5 establishes a checkpoint for 60S assembly during 40S maturation. RNA *25*, 1164–1176.

Kim, D., Paggi, J.M., Park, C., Bennett, C., and Salzberg, S.L. (2019). Graph-based genome alignment and genotyping with HISAT2 and HISAT-genotype. Nat. Biotechnol. *37*, 907–915.

Kiss-László, Z., Henry, Y., Bachellerie, J.P., Caizergues-Ferrer, M., and Kiss, T. (1996). Site-specific ribose methylation of preribosomal RNA: a novel function for small nucleolar RNAs. Cell *85*, 1077–1088.

Kornprobst, M., Turk, M., Kellner, N., Cheng, J., Flemming, D., Koš-Braun, I., Koš, M., Thoms, M., Berninghausen, O., Beckmann, R., et al. (2016). Architecture of the 90S pre-ribosome: a structural view on the birth of the eukaryotic ribosome. Cell *166*, 380–393.

Koš, M., and Tollervey, D. (2010). Yeast pre-rRNA processing and modification occur cotranscriptionally. Mol. Cell *37*, 809–820.

Krogan, N.J., Peng, W.T., Cagney, G., Robinson, M.D., Haw, R., Zhong, G., Guo, X., Zhang, X., Canadien, V., Richards, D.P., et al. (2004). High-definition macromolecular composition of yeast RNA-processing complexes. Mol. Cell *13*, 225–239.

Lamanna, A.C., and Karbstein, K. (2009). Nob1 binds the single-stranded cleavage site D at the 3′-end of 18S rRNA with its PIN domain. Proc. Natl. Acad. Sci. *106*, 14259–14264.

Lau, B., Cheng, J., Flemming, D., La Venuta, G., Berninghausen, O., Beckmann, R., and Hurt, E. (2021). Structure of the maturing 90S pre-ribosome in association with the RNA exosome. Mol. Cell *81*, 293–303.e4.

Lebaron, S., Segerstolpe, Å., French, S.L., Dudnakova, T., de lima Alves, F., Granneman, S., Rappsilber, J., Beyer, A.L., Wieslander, L., and Tollervey, D. (2013). Rrp5 binding at multiple sites coordinates Pre-rRNA processing and assembly. Mol. Cell *52*, 707–719.

Longtine, M.S., McKenzie, A., Demarini, D.J., Shah, N.G., Wach, A., Brachat, A., Philippsen, P., and Pringle, J.R. (1998). Additional modules for versatile and economical PCR-based gene deletion and modification in Saccharomyces cerevisiae. Yeast *14*, 953–961.

Ma, C., Wu, S., Li, N., Chen, Y., Yan, K., Li, Z., Zheng, L., Lei, J., Woolford, J.L., and Gao, N. (2017). Structural snapshot of cytoplasmic pre-60S ribosomal particles bound with Nmd3, Lsg1, Tif6 and Reh1. Nat. Struct. Mol. Biol. *24*, 214–220.

Martin, M. (2011). Cutadapt removes adapter sequences from high-throughput sequencing reads. EMBnet.Journal *17*, 10.

Matsuo, Y., Granneman, S., Thoms, M., Manikas, R.G., Tollervey, D., and Hurt, E. (2014). Coupled GTPase and remodelling ATPase activities form a checkpoint for ribosome export. Nature *505*, 112–116.

Meyer, B., Wurm, J.P., Kötter, P., Leisegang, M.S., Schilling, V., Buchhaupt, M., Held, M., Bahr, U., Karas, M., Heckel, A., et al. (2011). The Bowen-Conradi syndrome protein Nep1 (Emg1) has a dual role in eukaryotic ribosome biogenesis, as an essential assembly factor and in the methylation of Ψ1191 in yeast 18S rRNA. Nucleic Acids Res. *39*, 1526–1537.

Miller, O.L., and Beatty, B.R. (1969). Visualization of nucleolar genes. Science *164*, 955–957.

Mitchell, P., Petfalski, E., Shevchenko, A., Mann, M., and Tollervey, D. (1997). The exosome: a conserved eukaryotic RNA processing complex containing multiple 3'→5' exoribonucleases. Cell *91*, 457–466.

Mnaimneh, S., Davierwala, A.P., Haynes, J., Moffat, J., Peng, W.T., Zhang, W., Yang, X., Pootoolal, J., Chua, G., Lopez, A., et al. (2004). Exploration of essential gene functions via titratable promoter alleles. Cell *118*, 31–44.

Mougey, E.B., O'Reilly, M., Osheim, Y., Miller, O.L., Beyer, A., and Sollner-Webb, B. (1993). The terminal balls characteristic of eukaryotic rRNA transcription units in chromatin spreads are rRNA processing complexes. Genes Dev. *7*, 1609–1619.

Nousbeck, J., Spiegel, R., Ishida-Yamamoto, A., Indelman, M., Shani-Adir, A., Adir, N., Lipkin, E., Bercovici, S., Geiger, D., van Steensel, M.A., et al. (2008).

Alopecia, neurological defects, and endocrinopathy syndrome caused by decreased expression of RBM28, a nucleolar protein associated with ribosome biogenesis. Am. J. Hum. Genet. *82*, 1114–1121.

Osheim, Y.N., French, S.L., Keck, K.M., Champion, E.A., Spasov, K., Dragon, F., Baserga, S.J., and Beyer, A.L. (2004). Pre-18S ribosomal RNA is structurally compacted into the SSU processome prior to being cleaved from nascent transcripts in Saccharomyces cerevisiae. Mol. Cell *16*, 943–954.

Parks, T.D., Leuther, K.K., Howard, E.D., Johnston, S.A., and Dougherty, W.G. (1994). Release of proteins and peptides from fusion proteins using a recombinant plant virus proteinase. Anal. Biochem. *216*, 413–417.

Pérez-Fernández, J., Román, Á., De Las Rivas, J., Bustelo, X.R., and Dosil, M. (2007). The 90S preribosome is a multimodular structure that is assembled through a hierarchical mechanism. Mol. Cell. Biol. *27*, 5414–5429.

Pertschy, B., Schneider, C., Gnädig, M., Schäfer, T., Tollervey, D., and Hurt, E. (2009). RNA helicase Prp43 and its co-factor Pfa1 promote 20 to 18 S rRNA processing catalyzed by the endonuclease Nob1. J. Biol. Chem. *284*, 35079–35091.

Pettersen, E.F., Goddard, T.D., Huang, C.C., Couch, G.S., Greenblatt, D.M., Meng, E.C., and Ferrin, T.E. (2004). UCSF Chimera - a visualization system for exploratory research and analysis. J. Comput. Chem. *25*, 1605–1612.

Piekna-Przybylska, D., Decatur, W.A., and Fournier, M.J. (2007). New bioinformatic tools for analysis of nucleotide modifications in eukaryotic rRNA. RNA *13*, 305–312.

Pratte, D., Singh, U., Murat, G., and Kressler, D. (2013). Mak5 and Ebp2 act together on early pre-60S particles and their reduced functionality bypasses the requirement for the essential pre-60S factor Nsa1. PLoS One *8*, 1–23.

Punjani, A., Rubinstein, J.L., Fleet, D.J., and Brubaker, M.A. (2017). CryoSPARC: algorithms for rapid unsupervised cryo-EM structure determination. Nat. Methods *14*, 290–296.

Purta, E., Kaminska, K.H., Kasprzak, J.M., Bujnicki, J.M., and Douthwaite, S. (2008). YbeA is the m3ψ methyltransferase RlmH that targets nucleotide 1915 in 23S rRNA. RNA *14*, 2234–2244.

Reichow, S.L., Hamma, T., Ferré-D'Amaré, A.R., and Varani, G. (2007). The structure and function of small nucleolar ribonucleoproteins. Nucleic Acids Res. *35*, 1452–1464.

Robinson, J.T., Thorvaldsdóttir, H., Winckler, W., Guttman, M., Lander, E.S., Getz, G., and Mesirov, J.P. (2011). Integrative genomics viewer. Nat. Biotechnol. *29*, 24–26.

Rosado, I.V., and De La Cruz, J. (2004). Npa1p is an essential trans-acting factor required for an early step in the assembly of 60S ribosomal subunits in Saccharomyces cerevisiae. RNA *10*, 1073–1083.

Rosado, I.V., Dez, C., Lebaron, S., Caizergues-Ferrer, M., Henry, Y., and de la Cruz, J. (2007). Characterization of Saccharomyces cerevisiae Npa2p (Urb2p) reveals a low-molecular-mass complex containing Dbp6p, Npa1p (Urb1p), Nop8p, and Rsa3p involved in early steps of 60S ribosomal subunit biogenesis. Mol. Cell. Biol. *27*, 1207–1221.

Sá-Moura, B., Kornprobst, M., Kharde, S., Ahmed, Y.L., Stier, G., Kunze, R., Sinning, I., and Hurt, E. (2017). Mpp10 represents a platform for the interaction of multiple factors within the 90S pre-ribosome. PLoS One *12*, e0183272.

Sanghai, Z.A., Miller, L., Molloy, K.R., Barandun, J., Hunziker, M., Chaker-Margot, M., Wang, J., Chait, B.T., and Klinge, S. (2018). Modular assembly of the nucleolar pre-60S ribosomal subunit. Nature *556*, 126–129.

Schattner, P., Decatur, W.A., Davis, C.A., Ares, M., Fournier, M.J., and Lowe, T.M. (2004). Genome-wide searching for pseudouridylation guide snoRNAs: analysis of the Saccharomyces cerevisiae genome. Nucleic Acids Res. *32*, 4281–4296.

Schimmang, T., Tollervey, D., Kern, H., Frank, R., and Hurt, E.C. (1989). A yeast nucleolar protein related to mammalian fibrillarin is associated with small nucleolar RNA and is essential for viability. EMBO J. *8*, 4015–4024.

Singh, S., Broeck, A.V., Miller, L., Chaker-Margot, M., and Klinge, S. (2021). Nucleolar maturation of the human small subunit processome. Science *80*, 373.

Strunk, B.S., Novak, M.N., Young, C.L., and Karbstein, K. (2012). A translation-like cycle is a quality control checkpoint for maturing 40S ribosome subunits. Cell *150*, 111–121.

Sun, C., and Woolford, J.L. (1994). The yeast NOP4 gene product is an essential nucleolar protein required for pre-rRNA processing and accumulation of 60S ribosomal subunits. EMBO J. *13*, 3127–3135.

Sun, Q., Zhu, X., Qi, J., An, W., Lan, P., Tan, D., Chen, R., Wang, B., Zheng, S., Zhang, C., et al. (2017). Molecular architecture of the 90S small subunit pre-ribosome. Elife *6*, e22086.

Szczepińska, T., Kutner, J., Kopczyński, M., Pawłowski, K., Dziembowski, A., Kudlicki, A., Ginalski, K., and Rowicka, M. (2014). Probabilistic approach to predicting substrate specificity of methyltransferases. PLoS Comput. Biol. *10*, e1003514.

Talkish, J., Campbell, I.W., Sahasranaman, A., Jakovljevic, J., and Woolford, J.L. (2014). Ribosome assembly factors Pwp1 and Nop12 are important for folding of 5.8S rRNA during ribosome biogenesis in Saccharomyces cerevisiae. Mol. Cell. Biol. *34*, 1863–1877.

Talkish, J., Biedka, S., Jakovljevic, J., Zhang, J., Tang, L., Strahler, J.R., Andrews, P.C., Maddock, J.R., and Woolford, J.L. (2016). Disruption of ribosome assembly in yeast blocks cotranscriptional pre-rRNA processing and affects the global hierarchy of ribosome biogenesis. RNA *22*, 852–866.

Taoka, M., Nobe, Y., Yamaki, Y., Yamauchi, Y., Ishikawa, H., Takahashi, N., Nakayama, H., and Isobe, T. (2016). The complete chemical structure of Saccharomyces cerevisiae rRNA: partial pseudouridylation of U2345 in 25S rRNA by snoRNA snR9. Nucleic Acids Res. *44*, 8951–8961.

Thomas, B.J., and Rothstein, R. (1989). Elevated recombination rates in transcriptionally active DNA. Cell *56*, 619–630.

Thoms, M., Thomson, E., Baßler, J., Gnädig, M., Griesel, S., and Hurt, E. (2015). The exosome is recruited to RNA substrates through specific adaptor proteins. Cell *162*, 1029–1038.

Thoms, M., Mitterer, V., Kater, L., Falquet, L., Beckmann, R., Kressler, D., and Hurt, E. (2018). Suppressor mutations in Rpf2–Rrs1 or Rpl5 bypass the Cgr1 function for pre-ribosomal 5S RNP-rotation. Nat. Commun. *9*, 1–13.

Thomson, E., and Tollervey, D. (2010). The final step in 5.8S rRNA processing is cytoplasmic in Saccharomyces cerevisiae. Mol. Cell. Biol. *30*, 976–984.

Tkaczuk, K.L., Dunin-Horkawicz, S., Purta, E., and Bujnicki, J.M. (2007). Structural and evolutionary bioinformatics of the SPOUT superfamily of methyltransferases. BMC Bioinformatics *8*, 73.

Tollervey, D., Lehtonen, H., Jansen, R., Kern, H., and Hurt, E.C. (1993). Temperature-sensitive mutations demonstrate roles for yeast fibrillarin in pre-rRNA processing, pre-rRNA methylation, and ribosome assembly. Cell *72*, 443–457.

Trendel, J., Schwarzl, T., Horos, R., Prakash, A., Bateman, A., Hentze, M.W., and Krijgsveld, J. (2019). The human RNA-binding proteome and its dynamics during translational arrest. Cell *176*, 391–403.e19.

Udem, S.A., and Warner, J.R. (1972). Ribosomal RNA synthesis in Saccharomyces cerevisiae. J. Mol. Biol. *65*, 227–242.

Ulbrich, C., Diepholz, M., Baßler, J., Kressler, D., Pertschy, B., Galani, K., Böttcher, B., and Hurt, E. (2009). Mechanochemical removal of ribosome biogenesis factors from nascent 60S ribosomal subunits. Cell *138*, 911–922.

Venema, J., and Tollervey, D. (1996). RRP5 is required for formation of both 18S and 5.8S rRNA in yeast. EMBO J. *15*, 5701–5714.

Wagner, T., Merino, F., Stabrin, M., Moriya, T., Antoni, C., Apelbaum, A., Hagel, P., Sitsel, O., Raisch, T., Prumbaum, D., et al. (2019). SPHIRE-crYOLO is a fast and accurate fully automated particle picker for cryo-EM. Commun. Biol. *2*, 1–13.

Watkins, N.J., and Bohnsack, M.T. (2012). The box C/D and H/ACA snoRNPs: key players in the modification, processing and the dynamic folding of ribosomal RNA. Wiley Interdiscip. Rev. RNA *3*, 397–414.

CellPress
OPEN ACCESS

Woolford, J.L., and Baserga, S.J. (2013). Ribosome biogenesis in the yeast Saccharomyces cerevisiae. Genetics *195*, 643–681.

Yang, J., Sharma, S., Watzinger, P., Hartmann, J.D., Kötter, P., and Entian, K.D. (2016). Mapping of complete set of ribose and base modifications of yeast rRNA by RP-HPLC and mung bean nuclease assay. PLoS One *11*, 1–18.

Zagorski, J., Tollervey, D., and Fournier, M.J. (1988). Characterization of an SNR gene locus in Saccharomyces cerevisiae that specifies both dispensible and essential small nuclear RNAs. Mol. Cell. Biol. *8*, 3282–3290.

Zhang, K. (2016). Gctf: real-time CTF determination and correction. J. Struct. Biol. *193*, 1–12.

Zhang, L., Wu, C., Cai, G., Chen, S., and Ye, K. (2016). Stepwise and dynamic assembly of the earliest precursors of small ribosomal subunits in yeast. Genes Dev. *30*, 718–732.

Zhou, D., Zhu, X., Zheng, S., Tan, D., Dong, M.-Q., and Ye, K. (2018). Cryo-EM structure of an early precursor of large ribosomal subunit reveals a half-assembled intermediate. Protein Cell *10*, 120–130.

Zivanov, J., Nakane, T., Forsberg, B.O., Kimanius, D., Hagen, W.J.H., Lindahl, E., and Scheres, S.H.W. (2018). New tools for automated high-resolution cryo-EM structure determination in RELION-3. Elife *7*, e42166.

Zivanov, J., Nakane, T., and Scheres, S.H.W. (2020). Estimation of high-order aberrations and anisotropic magnification from cryo-EM data sets in RELION-3.1. IUCrJ *7*, 253–267.

STAR★METHODS

KEY RESOURCES TABLE

REAGENT or RESOURCE	SOURCE	IDENTIFIER
Chemicals, peptides, and recombinant proteins		
Flag peptide (DYKDDDDK)	CASLO	N/A
TEV protease	(Parks et al., 1994)	N/A
SIGMA*FAST*	Sigma–Aldrich	S8830
Critical commercial assays		
ANTI-FlagM2 Affinity Gel	Sigma–Aldrich	A2220; RRID:AB_10063035
IgG–Sepharose 6 Fast Flow	GE Healthcare	17096902
SYBR Green II RNA gel stain	Sigma–Aldrich	S9305
RiboLock RNase inhibitor	Thermo Scientific	EO0381
T4 PNK	NEB	M0201
mirVana miRNA Isolation Kit, with phenol	Invitrogen	AM1560
DNase I (RNase-free)	NEB	M0303S
NEBNext Multiplex Small RNA Library Prep Set for Illumina (Set 1)	NEB	E7300S
Bioanalyzer High Sensitivity DNA Analysis	Agilent	5067-4626
Qubit RNA HS Assay Kit	Invitrogen	Q32855
Qubit dsDNA HS Assay Kit	Invitrogen	Q32854
MiniSeq High Output Reagent Kit	Illumina	FC-420-1003
Deposited Data		
Cryo-EM volume: Urb2-Noc1 *mak5* D333A pre-60S particle	This paper	EMD-14507
Cryo-EM volume: 90S particle/90S-pre-60S bipartite particle	This paper	EMD-14508
Illumina sequencing data	This paper	PRJEB48541
Original gels and Northern blots	This paper	https://dx.doi.org/10.17632/v6sv3hhb8t.1
Experimental Models: Organism/Strains		
(Saccharomyces cerevisiae) MATalpha *ade2-1, trp1-1, leu2-3,112, his3-11,15, ura3-1, can1-100*	(Thomas and Rothstein, 1989)	W303
TF38 Null (haploid), *NOP1*, *ura3*, *his3*	(Tollervey et al., 1993)	NOP1+
TF38 Null (haploid), *nop1::URA3*, *his3*, LEU2, pSB32-*nop1-4*	(Tollervey et al., 1993)	nop1-4
TF38 Null (haploid), *NOP1*, *ura3*, *his3*, *NSA3-FTpA::natNT2*	This paper	NOP1+, Nsa3-FTpA
TF38 Null (haploid), *nop1::URA3*, *his3*, LEU2, pSB32-*nop1-4*, *NSA3-FTpA::natNT2*	This paper	nop1-4, Nsa3-FTpA
W303, *URB2-TAP::HIS3*, *NOC1-Flag::natNT2*	This paper	Urb2-TAP Noc1-Flag
W303, *URB2-TAP::HIS3*, *NOP58-Flag::natNT2*	This paper	Urb2-TAP Nop58-Flag
W303, *URB2-TAP::HIS3*, *NSA3-Flag::natNT2*	This paper	Urb2-TAP Nsa3-Flag
W303, *NSA1-TAP::klURA3*, *Flag-YTM1::natNT2*	(Kater et al., 2017)	Nsa1-TAP Flag-Ytm1
W303, *NOC4-TAP::HIS3*, *DHR1-Flag::natNT2*	(Cheng et al., 2020)	Noc4-TAP Dhr1-Flag
W303, *NOP58-FTpA::natNT2*	This paper	Nop58-FTpA
W303, *ARX1-FTpA::natNT2*	(Thoms et al., 2018)	Arx1-FTpA
W303, *Kre33-FTpA::HIS3*	(Cheng et al., 2019)	Kre33-FTpA
W303, *snr190:: klTRP1*	This paper	snR190Δ
W303, *snr37::hphNT1*	This paper	snR37Δ
W303, *snr190::TRP*, *snr37::hphNT1*	This paper	snR190Δ snR37Δ
W303, *rsa3::natNT2*	This paper	Rsa3Δ
W303, *snr190:: klTRP1*, *rsa3::natNT2*	This paper	snR190Δ Rsa3Δ

(*Continued on next page*)

Continued

REAGENT or RESOURCE	SOURCE	IDENTIFIER
W303, *URB2-TAP*::*HIS3*, *NOC1-Flag*::*natNT2*, *snr190*:: *klTRP1*	This paper	Urb2-TAP Noc1-Flag snR190Δ
W303, *URB2-TAP*::*HIS3*, *NOC1-Flag*::*natNT2*, *snr37*::*hphNT1*	This paper	Urb2-TAP Noc1-Flag snR37Δ
W303, *URB2-TAP*::*HIS3*, *NOC1-Flag*::*natNT2*, *snr190*:: *klTRP1*, *snr37*::*hphNT1*	This paper	Urb2-TAP Noc1-Flag snR190Δ snR37Δ
W303, *NSA3-FTpA*::*natNT2*	This paper	Nsa3-FTpA
W303, *NSA3-FTpA*::*natNT2*, *snr190*:: *klTRP1*	This paper	Nsa3-FTpA snR190Δ
W303, *NSA3-FTpA*::*HIS3*, *rsa3*::*natNT2*	This paper	Nsa3-FTpA Rsa3Δ
W303, *NSA3-FTpA*::*HIS3*, *snr190*:: *klTRP1*, *rsa3*::*natNT2*	This paper	Nsa3-FTpA snR190Δ Rsa3Δ
W303, *UPA1-FTpA*::*natNT2*	This paper	Upa1-FTpA
W303, *UPA2-FTpA*::*natNT2*	This paper	Upa2-FTpA
Recombinant DNA		
p.GAL, *MAK5*, *LEU2*, ARS/ CEN, *AmpR*	This paper	YCplac111-GAL-*MAK5*
p.GAL, *mak5 D333A*, *LEU2*, ARS/ CEN, *AmpR*	This paper	YCplac111-GAL-*mak5 D333A*
Software and algorithms		
MaxQuant	(Cox and Mann, 2008)	https://www.maxquant.org
UCSF Chimera	(Pettersen et al., 2004)	http://www.cgl.ucsf.edu/chimera
ChimeraX	(Goddard et al., 2018)	https://www.rbvi.ucsf.edu/chimerax
Fastqc	Andrews, S. (2010). FastQC: A Quality Control Tool for High Throughput Sequence Data [Online]	https://www.bioinformatics.babraham.ac.uk/projects/fastqc/
Cutadapt	(Martin, 2011)	https://cutadapt.readthedocs.io/en/stable/installation.html
Hisat2	(Kim et al., 2019)	http://daehwankimlab.github.io/hisat2/
Samtools	(Danecek et al., 2021)	http://www.htslib.org/
R-studio	RStudio Team (2020). RStudio: Integrated Development for R. RStudio, PBC, Boston, MA	https://www.rstudio.com/
ggplot2 (R package)	Wickham H (2016). ggplot2: Elegant Graphics for Data Analysis. Springer-Verlag New York. ISBN 978-3-319-24277-4	https://ggplot2.tidyverse.org
IGV	(Robinson et al., 2011)	https://software.broadinstitute.org/software/igv/
CrYOLO	(Wagner et al., 2019)	http://sphire.mpg.de
RELION 3.1.2	(Zivanov et al., 2018) (Zivanov et al., 2020)	https://github.com/3dem/relion
CryoSPARC v3.2.0	(Punjani et al., 2017)	http://www.cryosparc.com/
Gctf	(Zhang, 2016)	https://www2.mrc-lmb.cam.ac.uk/research/locally-developed-software/zhang-software/

RESOURCE AVAILABILITY

Lead contact

Further information and requests for resources and reagents should be directed to and will be fulfilled by the Lead Contact, Ed Hurt (ed.hurt@bzh.uni-heidelberg.de).

Materials availability

This study did not generate new unique reagents. Plasmids and strains are available from the authors upon request.

Data and code availability

Cryo-EM volumes generated in this study have been deposited with accession codes EMD-14507 and EMD-14508. Raw Illumina sequencing data have been deposited at the European Nucleotide Archive ENA with project number: PRJEB48541. All raw data for Coomassie-stained SDS-polyacrylamide gels and northern blots have been deposited with Mendeley and can be accessed at https://doi.org/10.17632/v6sv3hhb8t.1.

This paper does not report original code. Additional information required to reanalyze the data reported in this paper is available from the Lead Contact upon request.

EXPERIMENTAL MODEL AND SUBJECT DETAILS

Yeast strains

The genotypes of the *Saccharomyces cerevisiae* strains used are listed in the Key Resource Table.

Bacterial strains

The *E. coli* DH5a strain was used for plasmid construction.

METHOD DETAILS

Yeast strains

All yeast strains generated and used in this work are listed in the Key Resource Table. Genomic integration of tags and gene disruptions was performed using standard techniques (Janke et al., 2004; Longtine et al., 1998). The strains were verified by western blotting using antibodies against tagged proteins, colony PCR, and/or sequencing.

To construct an *snR190Δ* strain, we took into consideration that snR190 and U14 are transcribed together as a dicistronic precursor transcript (Chanfreau et al., 1998). We used PCR to create a construct with an 80-nucleotide deletion in snR190 (*snR190Δ*) followed by U14 and TRP marker (*snR190Δ*-U14-native terminator-TRP). The W303 yeast strain was then transformed with this construct to replace genomic snR190-U14 by homologous recombination. The transformed strain was confirmed by colony PCR and sequencing.

Tandem affinity purification of pre-ribosomal particles

Yeast strains expressing single-bait proteins tagged with Flag-TEV-protA (FTpA) or strains expressing split tags—one protein with calmodulin-TEV-protA (TAP) and another with Flag—were cultured at 30°C and harvested at an $OD_{600\ nm}$ value of 2.0–3.0. Cells were lysed in purification buffer (100 mM NaCl, 5 mM $MgCl_2$, 50 mM Tris-HCl, pH 7.5, 5% (v/v) glycerol, 0.1% (v/v) IGEPAL CA-630, and 1 mM dithiothreitol) supplemented with protease inhibitor cocktail (SIGMA*FAST*) and RiboLock RNase inhibitor. Lysis was performed mechanically either in a bead beater (Fritsch) using glass beads or in a cryogenic mill (Retsch MM400), and lysates were pre-cleared by centrifugation at 4°C for 10 min at 5000 rpm then for 20 min at 17,000 rpm. For the first affinity step, the supernatants were incubated with pre-equilibrated IgG Sepharose 6 Fast Flow affinity resin (GE Healthcare) for at least 2.5 h at 4°C, which was then washed with purification buffer. The resin was collected and bound proteins were eluted from it by cleaving with TEV protease at 16°C for 1.5 h. For the second affinity step, TEV eluates were incubated with pre-equilibrated Anti-Flag M2 Affinity Gel (Sigma–Aldrich) for 1.5 h at 4°C, which was then washed with purification buffer. Then, bound proteins were eluted from the anti-Flag beads with Flag peptide (final concentration 300 μg/mL). Flag eluates were analyzed by on 4–12% polyacrylamide gels (NuPAGE, Invitrogen) with colloidal Coomassie staining (Roti Blue, Roth).

For the *Urb2–TAP Noc1-Flag* YCplac111-GAL-*mak5* D333A strain, the cells were cultured at 30°C in SDC-Leu (glucose) medium to early logarithmic phase then shifted to YPG (galactose) medium and grown for 8 h to induce *mak5* D333A. Then, affinity purification of Urb2-TAP Noc1-Flag particles was performed as described above with final elution from the anti-flag beads using Flag peptide (final concentration 300 μg/mL) in the following buffer 100 mM NaCl, 5 mM $MgCl_2$, 50 mM Tris-HCl, pH 7.5 and 5% (v/v) glycerol for negative-stain EM analysis. For cryo-EM analysis, the final elution was done using Flag peptide (final concentration 300 μg/mL) in the following buffer 100 mM NaCl, 5 mM $MgCl_2$, 50 mM Tris-HCl, pH 7.5 and 0.05% Nikkol.

RNA extraction and analysis

RNA was extracted from the Flag eluates of affinity-purified pre-ribosomes using phenol–chloroform extraction followed by precipitation with ethanol. For detection of snoRNAs, extracted RNA samples were resolved on 8% polyacrylamide/8 M urea gels. For RNA staining, the gels were incubated for 30 min with SYBR Green II RNA gel stain (Sigma–Aldrich) using a 1:5000 dilution in 1× TBE. For northern blot analysis, the RNA was transferred to a Hybond-N^+ nylon membrane (GE Healthcare) and UV-crosslinked. The following oligos (5′-labeled with ^{32}P) were used for detecting snR190, snR37, and U3, respectively: CGTCATGGTCGAATCGG, AAGCTCCTCA TCACTCACAC, and GGTTATGGGACTCATCA.

Sucrose gradient centrifugation

The Flag eluates of affinity purified pre-ribosomes were loaded on a 10–40% (w/v) linear sucrose gradient (prepared in buffer containing 100 mM NaCl, 5 mM $MgCl_2$, 50 mM Tris-HCl, pH 7.5) and centrifuged at 27,000 rpm for 16 h at 4°C. After centrifugation,

fractions were collected and an equal volume from each was used for RNA extraction and northern blotting (in case of Urb2-Nop58). The remainder of each fraction was precipitated with 10% TCA, resuspended in SDS sample buffer, and analyzed on 4–12% polyacrylamide gels (NuPAGE, Invitrogen) with colloidal Coomassie staining.

Mass spectrometry

Major bands from Coomassie-stained gels were individually excised and identified by MALDI-TOF mass spectrometry. Semiquantitative mass spectrometry was performed at the FingerPrints Proteomics Facility (University of Dundee, UK), where the proteins were identified by 1D nLC–ESI-MS/MS. Raw data was analyzed using MaxQuant software (Cox and Mann, 2008).

Electron microscopy and image processing

Negative-stain EM of isolated pre-ribosomal particles was performed essentially as described by (Lau et al., 2021). For cryo-EM, the Urb2–Noc1 mak5 D333A sample was snap-cooled in buffer containing 50 mM Tris-HCl pH 7.5, 100 mM NaCl, 5 mM $MgCl_2$ and 0.05% Nikkol. Three microliters of the sample were pipetted onto Quantifoil R 2/1 holey carbon grids, which were glow-discharged for 45 s using the PELCO easiGlow system. The grids were blotted for 5 s using a blot force of 10 at 100% humidity using a Vitrobot Mark IV (Thermo Fisher Scientific) operated at 4°C, and immediately plunge-frozen in liquid ethane cooled with liquid nitrogen. Grids were mounted onto auto-grids and imaged using a Thermo Scientific Glacios cryo-transmission electron microscope equipped with a Falcon IIIEC detector and operated at an acceleration voltage of 200 kV. One thousand and one movies were recorded in nanoprobe mode with parallel illumination at a nominal magnification of 45,000 and a spot size of 4 in linear mode. The calibrated object pixel size was 3.17 Å. Micrographs were acquired using dose fractionation to record 119 frames per exposure with a dose rate of 1 e per $Å^2$ per frame.

Particles were picked with CrYOLO (Wagner et al., 2019) and images were processed with the RELION 3.1.2 (Zivanov et al., 2018, 2020) and CryoSPARC v3.2.0 (Punjani et al., 2017) software packages. In brief, movie stacks were motion-corrected using MotionCor2 with 5 × 5 as the number of patches, and estimation of contrast transfer function was performed on the motion-corrected micrographs with Gctf (Zhang, 2016).

For particle picking, particles from 14 micrographs were manually picked with the CrYOLO box manager and used to refine the general model. A total number of 310,374 particles were picked, extracted using a box size of 180 × 180 pixels, and subjected to 2D classification in CryoSPARC. Good 2D classes were selected and the corresponding 69,486 particles were used for subsequent ab initio reconstruction with three classes. The resulting volumes were used as references for heterogeneous refinement with all picked particles. Homogeneous refinement was performed for the resulting pre-60S and 90S classes following re-extraction (box size 200 × 200) and 3D classification in RELION 3.1.2. Three-dimensional classification was performed on the pre-60S class, with image alignment and an angular sampling interval of 7.5°; no alignment was performed for the 90S class. The good pre-60S class and the 90S class were refined and local filtered with CryoSPARC. Furthermore, the 90S particles were re-extracted with a box size of 450 × 450 pixels and 3D refinement was performed with a mask of 1200 Å, which led to the reconstituted 90S connected to a large and flexible extra density. The particles were iteratively re-extracted, centered, and refined, allowing large shifts, and the final homogeneous refinement and local filtering was performed with CryoSPARC. ChimeraX was used to display cryo-EM structures and models (Goddard et al., 2018).

Illumina sequencing of RNA

RNA was extracted from the selected particles (Urb2–Nop58, Urb2–Noc1) using a mirVana miRNA isolation kit, following the manufacturer's protocol for enriching for small RNAs. After extraction, RNA was quantified using a Qubit RNA HS assay kit and used as input for library preparation with the NEBNext Small RNA Library Prep Set for Illumina, following the manufacturer's instructions. Libraries were loaded on a MiniSeq and sequenced in single-end mode with reads of 150 nts length. To generate the final Log2 fold-change values, raw reads were quality checked and processed using in-house scripts. Raw data is available at ENA (project: PRJEB48541; secondary accession: ERP132922).

QUANTIFICATION AND STATISTICAL ANALYSIS

See Method details on cryo-electron microscopy and image processing, and for mass spectrometry label-free quantification (LFQ values). No further statistical analysis was used in this study.

Cell Reports, Volume *39*

Supplemental information

Emergence of the primordial pre-60S

from the 90S pre-ribosome

Sherif Ismail, Dirk Flemming, Matthias Thoms, José Vicente Gomes-Filho, Lennart Randau, Roland Beckmann, and Ed Hurt

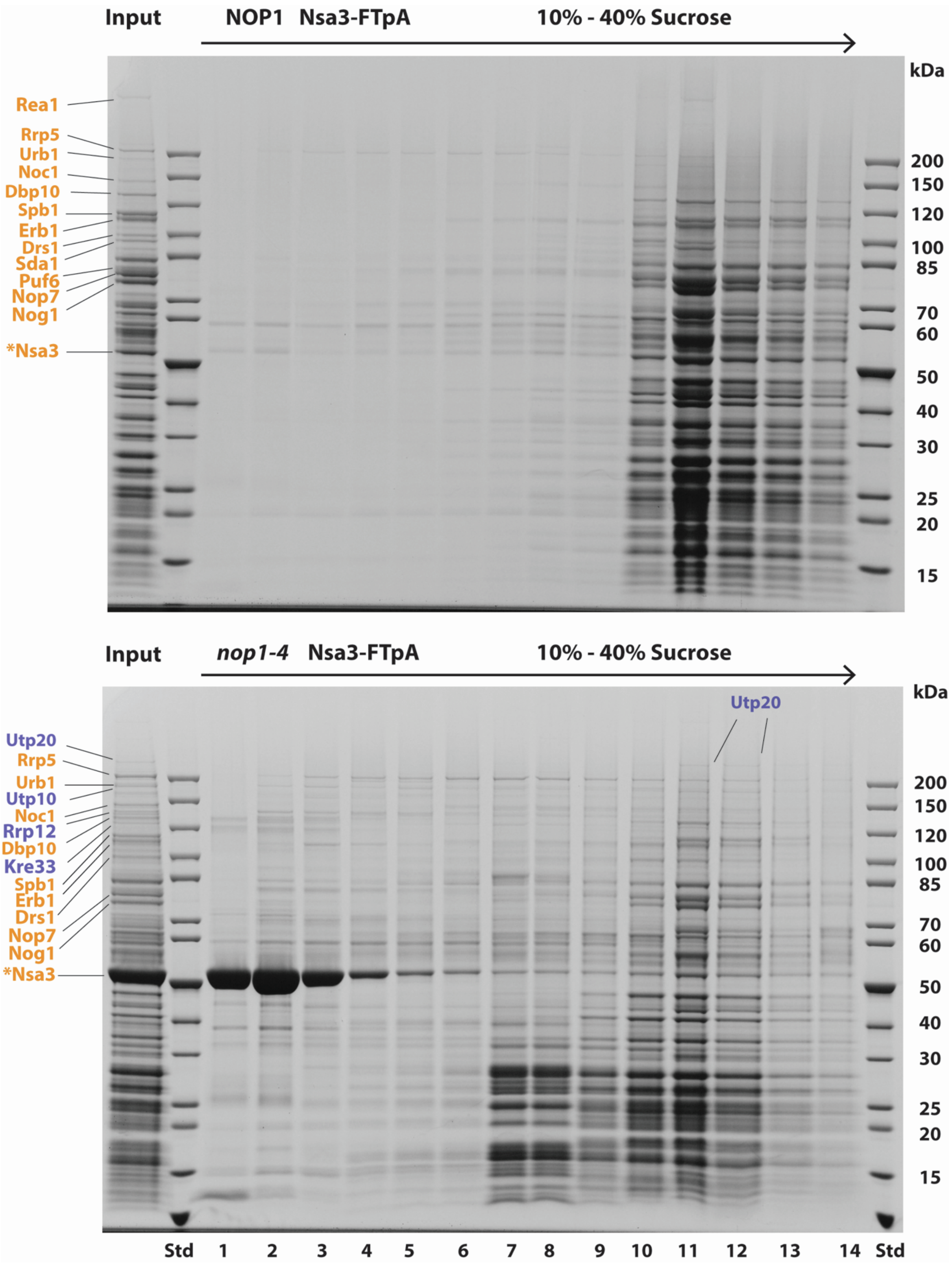

Figure S1. Sucrose Gradient Analysis of Nsa3 Pre-60S Particles from the *nop1-4* Mutant, Related to Figure 1

Affinity-purified Nsa3 particles from wild-type *NOP1* and *nop1-4* cells were fractionated by sucrose gradient centrifugation (10–40% sucrose) (w/v). 14 fractions were collected from each

gradient, which were TCA-precipitated and resuspended in SDS-sample buffer. The fractions were analyzed by 4–12% gradient SDS-PAGE and stained with Coomassie. Pre-60S assembly factors are colored in orange and 90S factors are colored in blue. The bait protein Nsa3 is indicated by an asterisk.

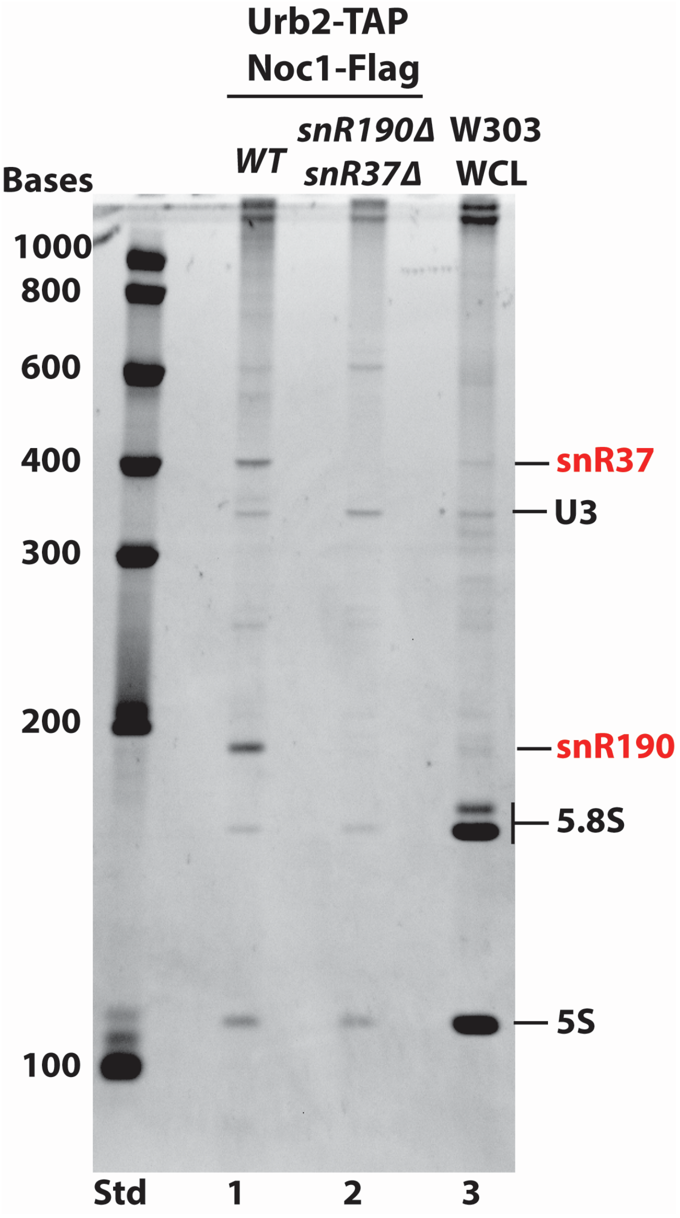

Figure S2. snR190 and snR37 are Absent from the Urb2–Noc1 Particle When Isolated from *snR190Δ snR37Δ* Double-Deletion Strain, Related to Figure 3 and 4

Polyacrylamide/urea gel electrophoresis and SYBR Green staining of RNA extracted from the final eluates of affinity-purified Urb2-TAP–Noc1-Flag, derived from the wild-type or *snR190Δ snR37Δ* double-deletion strains. RNA of a whole cell lysate (WCL) from strain W303 was also loaded on the 8% polyacrylamide/urea gel, which was stained with SYBR Green to detect the snR37 and snR190 bands, which are indicated in red on the right.

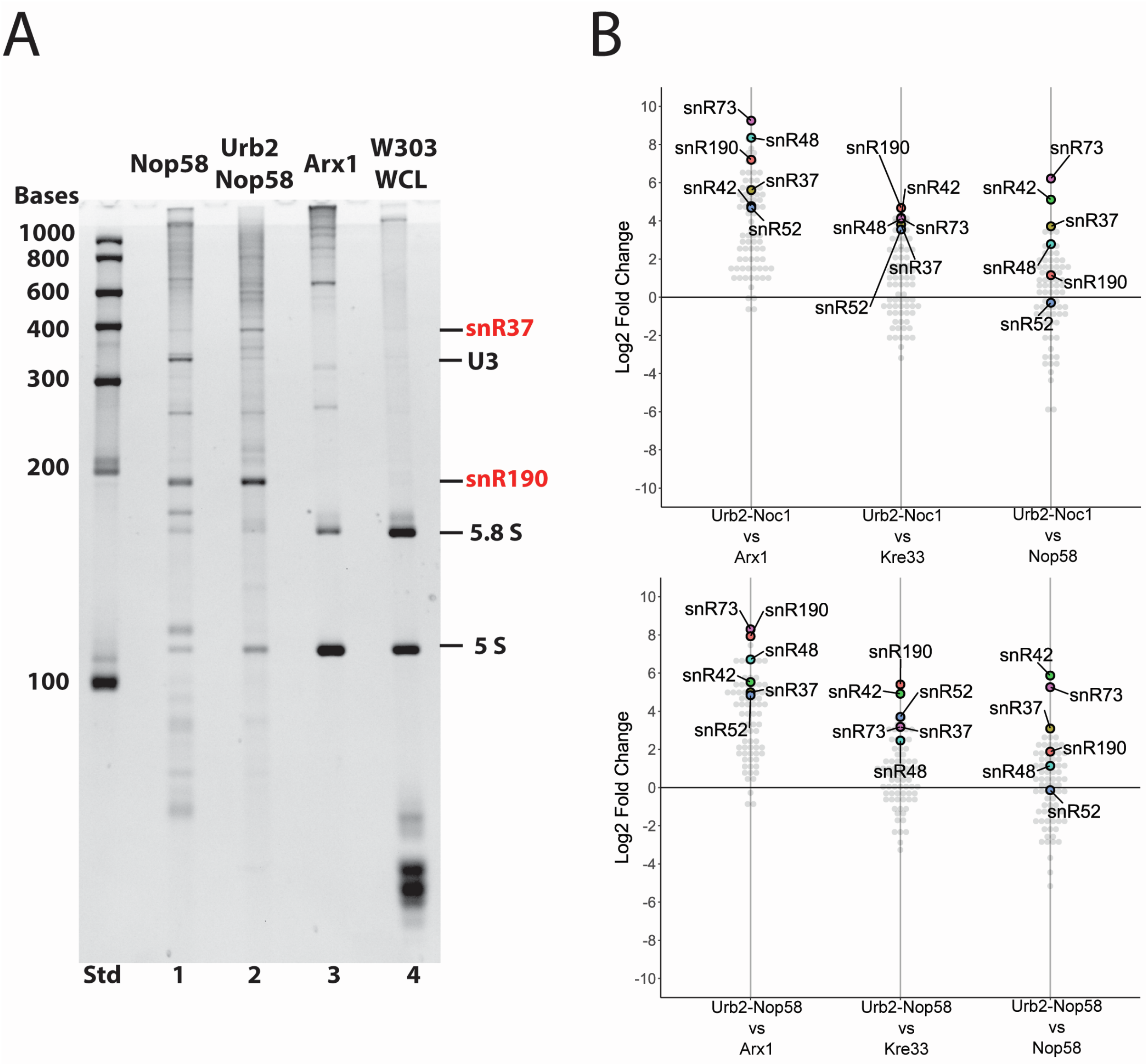

Figure S3. Identification of the snoRNAs Co-enriched in the Affinity-Purified Primordial Pre-60S Particles, Related to Figure 3

(A) SYBR Green staining of the 8% polyacrylamide/urea gel, on which was separated RNA extracted from Nop58-FTpA, Urb2-TAP–Nop58-Flag, and Arx1-FTpA affinity-purified particles, in comparison to total RNA from whole-cell lysate (WCL) of the wild-type W303 strain. Nop58 was used to reveal the pool of C/D box snoRNAs, whereas Urb2–Nop58 was used to show the snoRNA pool enriched in the primordial pre-60S.

(B) Illumina RNA sequencing of RNA extracted from Urb2-TAP Noc1-Flag, Urb2-TAP Nop58-Flag, Nop58-FTpA, Kre33-FTpA, and Arx1-FTpA final eluates. The Log2 Fold change of Illumina read counts was calculated for Urb2-Noc1 and Urb2-Nop58 preparations in comparison to Arx1, Kre33 and Nop58 samples (see Table S3 for further details).

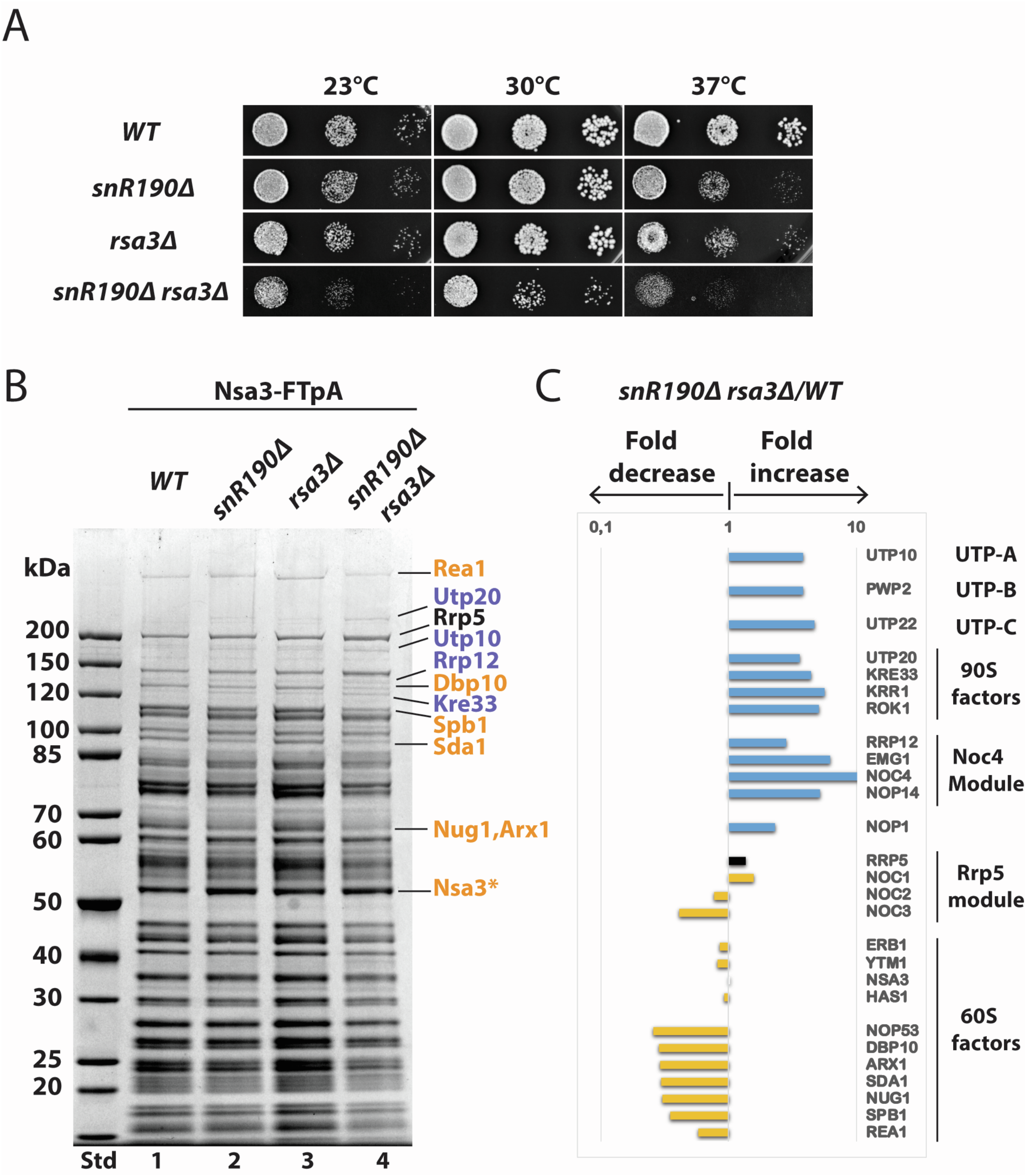

Figure S4. Synthetically Enhanced Growth Defect between snR190 and the Urb1 Module Factor Rsa3, Related to Figure 4

(A) Dot-spot growth analysis of wild-type W303 (WT), *snR190Δ*, *rsa3Δ*, and *snR190Δ rsa3Δ* strains. Tenfold serial dilutions were spotted onto YPD plates. The plates were incubated at 23, 30, or 37°C for 2 days.

(B) Affinity purification of Nsa3-FTpA from wild-type W303, *snR190Δ*, *rsa3Δ*, and *snR190 Δ rsa3Δ* strains. Flag eluates were analyzed on a 4–12% gradient SDS-PAGE gel and stained with Coomassie. The major bands labeled on the right were identified by mass spectrometry.

The 90S factors are colored in blue and pre-60S factors are colored in orange. The bridging factor Rrp5 is colored in black. The Nsa3 bait is indicated by an asterisk.
(C) Semiquantitative mass spectrometry analysis of the Nsa3-FTpA eluates in panel B. The label-free quantification values (normalized to Nsa3) derived from the *snR190Δ rsa3Δ* strain were divided by those obtained for the WT particles, which represents fold change in 90S (blue bars) and pre-60S (orange bars) assembly factors. For the whole data set of the mass spectrometry analysis, see Table S5.

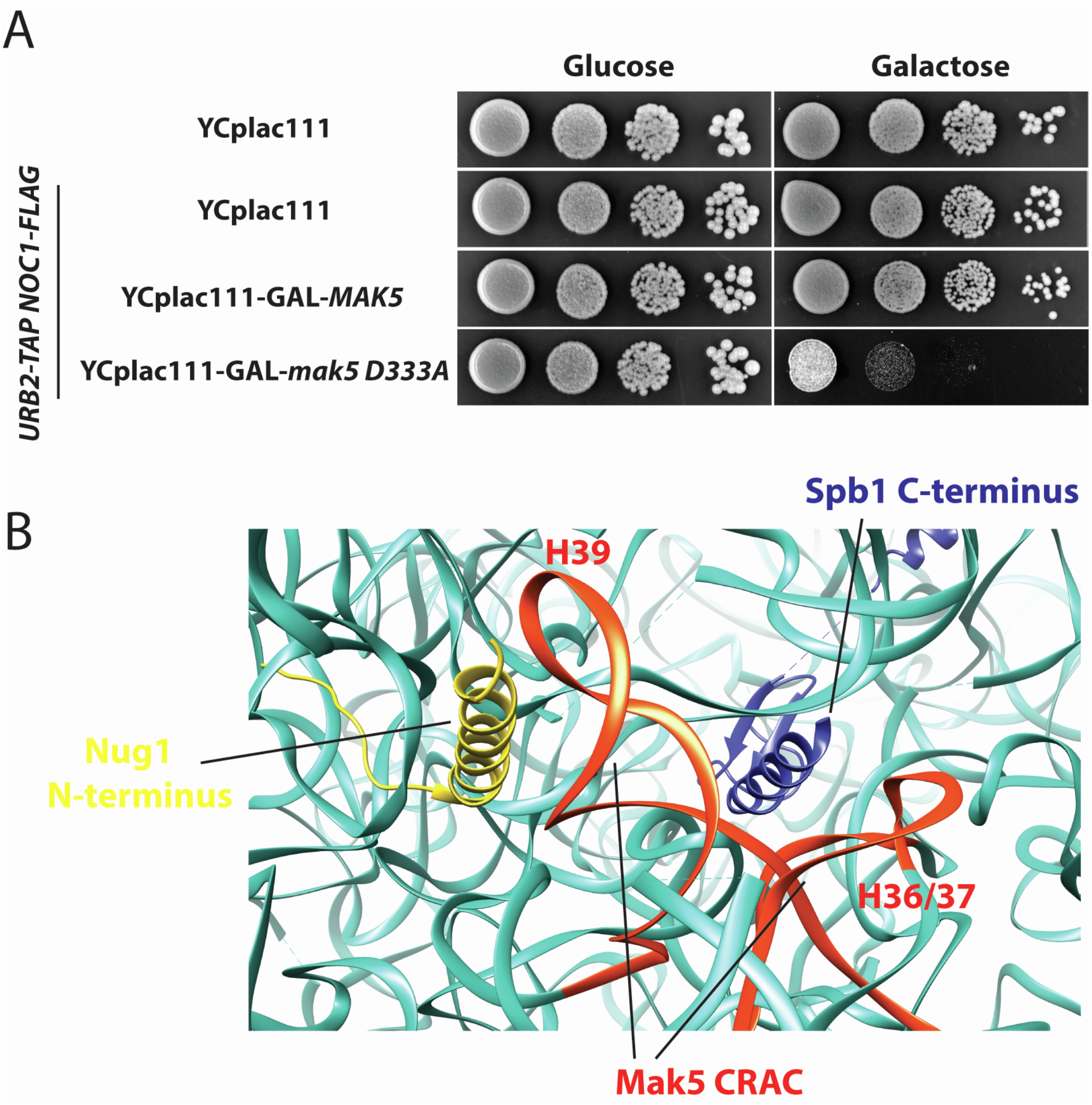

Figure S5. Dominant-Negative Mak5 D333A Walker B Mutant and Mak5-Binding Site in the Pre-60S Particles, Close to the N Terminus of Nug1 and the C Terminus of Spb1, Related to Figure 6

(A) Dot-spot growth analysis of the dominant-negative phenotype of *mak5 D333A* overexpression in the Urb2-TAP Noc1-Flag background. Tenfold serial dilutions of the indicated cells were spotted onto SDC−Leu (glucose) and SGC−Leu (galactose) plates. The plates were incubated at 30°C for 3 days.
(B) Nsa1–Ytm1 cryo-EM structure state D (PDB ID: 6EM5) with indicated CRAC crosslinking sites of Mak5 (Brüning et al., 2018) at helices H36/37 and H39 (Red). In addition, the nearby Nug1 N terminus (yellow) and Spb1 C terminus (blue) are highlighted.

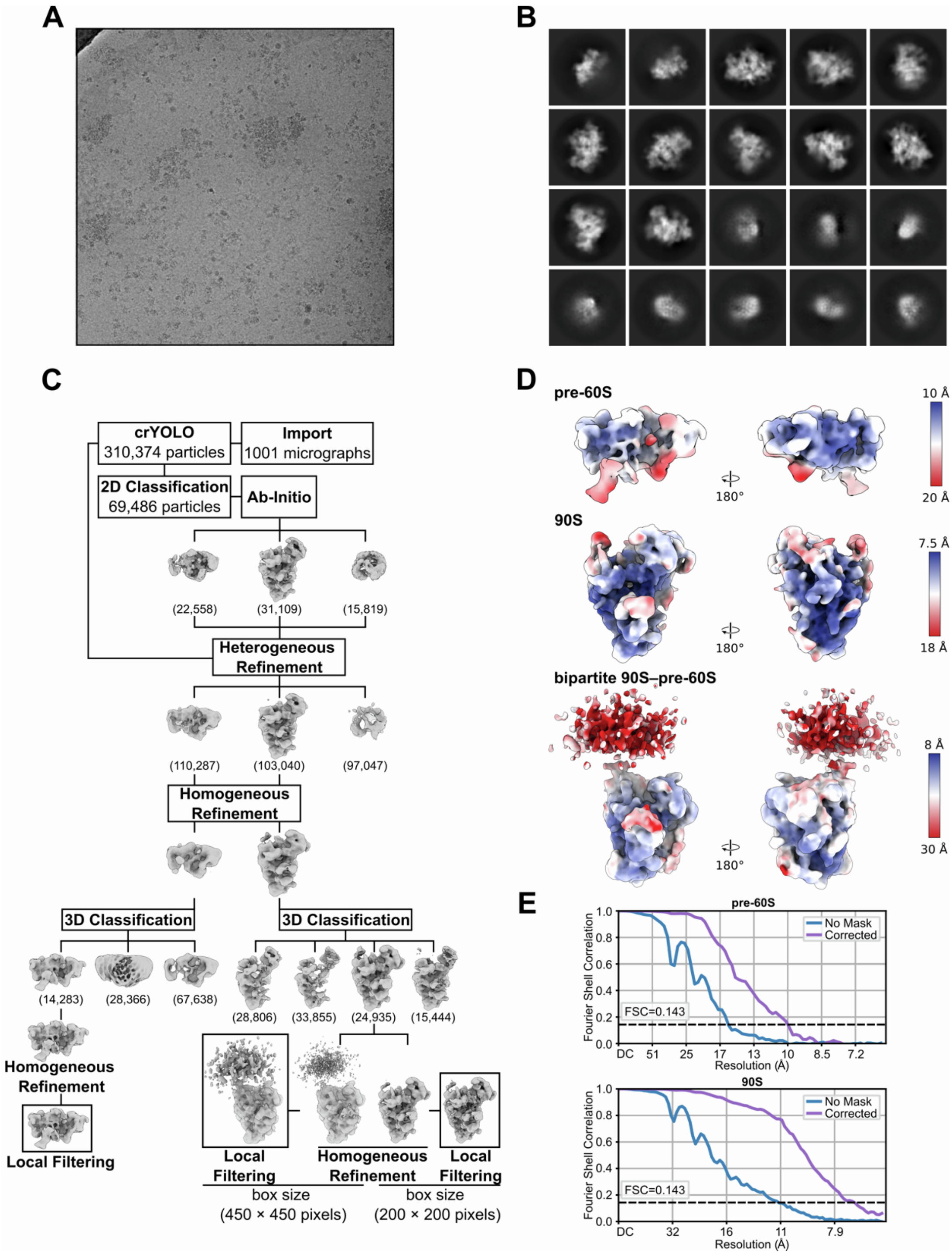

Figure S6. Sorting Scheme for the Urb2–Noc1 *mak5* D333A Particles, Related to Figure 7

(A, B) Representative electron micrograph (A) and selected 2D classification averages of the Urb2–Noc1 preparation isolated from the dominant-negative *mak5* D333A mutant (B).
(C) Cryo-EM processing scheme. Particle numbers are indicated in parentheses.
(D) The final 3D reconstructions after local filtering and colored according to their local resolution.
(E) Fourier shell correlation curves of the final pre-60S and 90S reconstructions. The FSC thresholds at 0.143 are indicated as dashed lines.

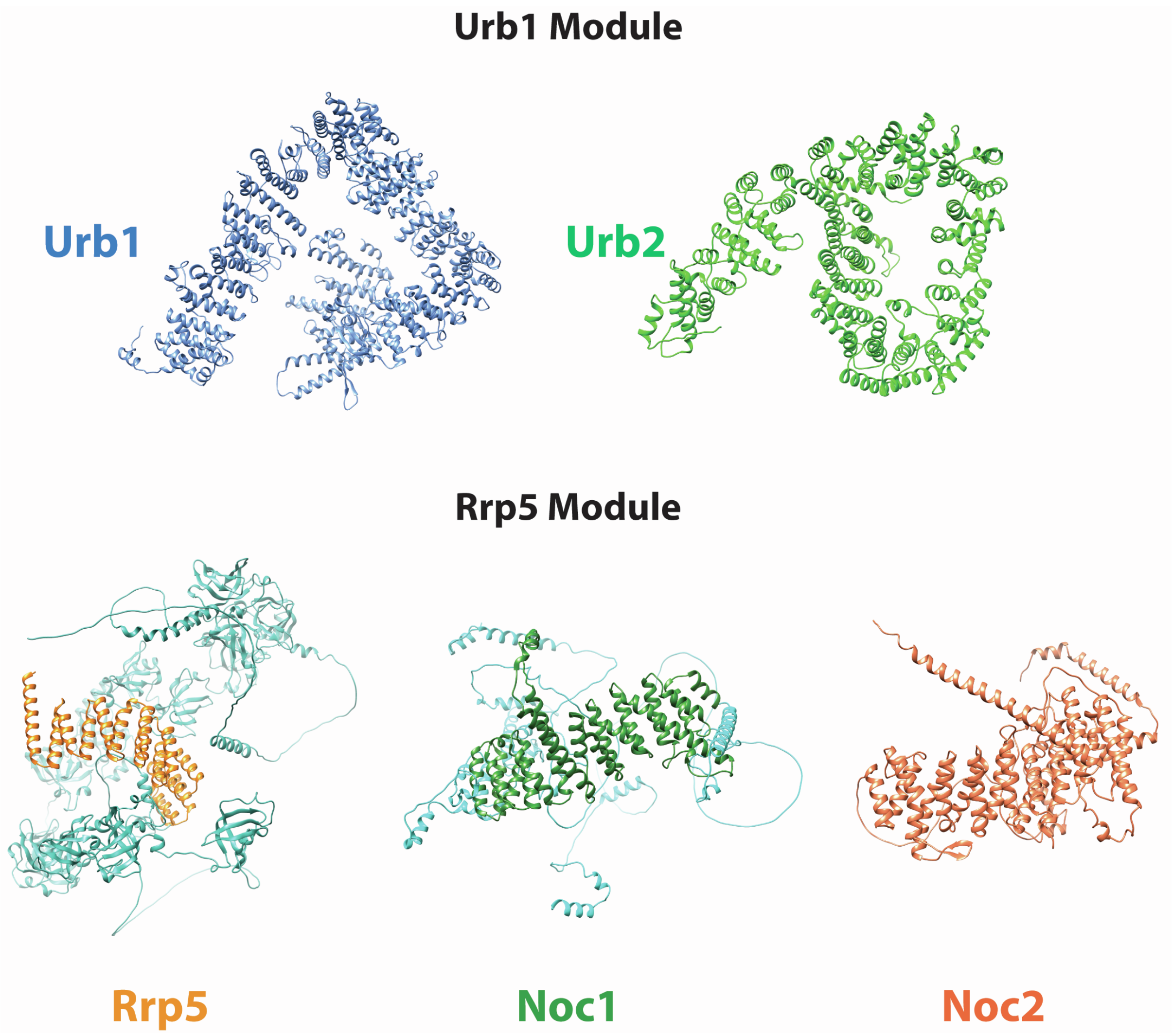

Figure S7. Structure prediction of Urb1 and Rrp5 module members, Related to Figure 7

Structure prediction of ribosome assembly factors of the Urb1 and Rrp5 modules using AlphaFold (Jumper et al., 2021). The α-helical repeats of Urb1 (light blue; code number: P34241), Urb2 (light green, code number: P47108), Rrp5 (orange, code number: Q05022), Noc1 (dark green, code number: Q12176) and Noc2 (coral, code number: P39744) forming α-solenoid folds are depicted. Beside the α-helical repeats in the C-terminal half (orange), Rrp5 contains 12 S1-like domains in row in its N-terminal part (greenish blue).
